# Enhanced cell survival in prepubertal testicular tissue cryopreserved with membrane lipids and antioxidants rich cryopreservation medium

**DOI:** 10.1007/s00441-024-03930-6

**Published:** 2024-11-25

**Authors:** Reyon Dcunha, Anjana Aravind, Smitha Bhaskar, Sadhana Mutalik, Srinivas Mutalik, Sneha Guruprasad Kalthur, Anujith Kumar, Padmaraj Hegde, Satish Kumar Adiga, Yulian Zhao, Nagarajan Kannan, Thottethodi Subrahmanya Keshava Prasad, Guruprasad Kalthur

**Affiliations:** 1 Division of Reproductive Genetics, Department of Reproductive Science, Kasturba Medical College, Manipal, Manipal Academy of Higher Education, Manipal, 576104 Karnataka India; 2 Center for Systems Biology and Molecular Medicine, Yenepoya Research Centre, Yenepoya (Deemed to be University), Mangalore, 575018 Karnataka India; 3 Manipal Institute of Regenerative Medicine, Manipal Academy of Higher Education, Allalasandra, Yelahanka, Bengaluru, 560065 Karnataka India; 4 Department of Pharmaceutics, Manipal College of Pharmaceutical Sciences, Manipal Academy of Higher Education, Manipal, 576104 Karnataka India; 5 Department of Anatomy, Kasturba Medical College, Manipal, Manipal Academy of Higher Education, Manipal, 576104 Karnataka India; 6 Department of Urology, Kasturba Medical College, Manipal, Manipal Academy of Higher Education, Manipal, 576104 Karnataka India; 7 Centre of Excellence in Clinical Embryology, Department of Reproductive Science, Kasturba Medical College, Manipal, Manipal Academy of Higher Education, Manipal, 576104 Karnataka India; 8 Department of Obstetrics and Gynecology and Department of Laboratory Medicine and Pathology, Mayo Clinic, Rochester, MN 55905 USA; 9 Division of Experimental Pathology and Laboratory Medicine, Department of Laboratory Medicine and Pathology, Mayo Clinic, Rochester, MN 55905 USA; 10 Center for Regenerative Medicine, Mayo Clinic, Rochester, MN 55905 USA; 11 Mayo Clinic Cancer Center, Mayo Clinic, Rochester, MN 55905 USA; 12 Division of Reproductive Biology, Department of Reproductive Science, Kasturba Medical College, Manipal, Manipal Academy of Higher Education, Manipal, 576104 Karnataka India

**Keywords:** Spermatogonial germ cells, Proteomics, Fertility preservation, Oncofertility, Membrane integrity

## Abstract

The present study explores the advantages of enriching the freezing medium with membrane lipids and antioxidants in improving the outcome of prepubertal testicular tissue cryopreservation. For the study, testicular tissue from Swiss albino mice of prepubertal age group (2 weeks) was cryopreserved by slow freezing method either in control freezing medium (CFM; containing DMSO and FBS in DMEM/F12) or test freezing medium (TFM; containing soy lecithin, phosphatidylserine, phosphatidylethanolamine, cholesterol, vitamin C, sodium selenite, DMSO and FBS in DMEM/F12 medium) and stored in liquid nitrogen for at least one week. The tissues were thawed and enzymatically digested to assess viability, DNA damage, and oxidative stress in the testicular cells. The results indicate that TFM significantly mitigated freeze–thaw-induced cell death, DNA damage, and lipid peroxidation compared to tissue cryopreserved in CFM. Further, a decrease in *Cyt C*, *Caspase-3,* and an increase in Gpx*4* mRNA transcripts were observed in tissues frozen with TFM. Spermatogonial germ cells (SGCs) collected from tissues frozen with TFM exhibited higher cell survival and superior DNA integrity compared to those frozen in CFM. Proteomic analysis revealed that SGCs experienced a lower degree of freeze–thaw-induced damage when cryopreserved in TFM, as evident from an increase in the level of proteins involved in mitigating the heat stress response, transcriptional and translational machinery. These results emphasize the beneficial role of membrane lipids and antioxidants in enhancing the cryosurvival of prepubertal testicular tissue offering a significant stride towards improving the clinical outcome of prepubertal testicular tissue cryopreservation.

**Supplementary Information:**

The online version contains supplementary material available at 10.1007/s00441-024-03930-6.

## Introduction

Childhood cancer remains a significant contributor to mortality among children aged 5–14 years (Kyu et al. 2018; Liu et al. 2022). While remarkable progress in cancer treatment has led to an increased survival rate of up to 80% in developed countries (Hudson 2010), long-term health consequences including infertility in adulthood remain a major medical challenge (Green et al. 2010; Brannigan 2014; Wallace et al. 2014) due to the gonadotoxic nature of cancer treatments (Wallace et al. 2014; Allen et al. 2018). The spermatogonial germ cells (SGCs) in prepubertal boys are susceptible to chemotherapy and/or radiotherapy-induced damage, exposure to which can potentially result in temporary or permanent azoospermia (Green et al. 2010; Meistrich 2013). Considering the uncertainty about their future fertility potential, prepubertal boys diagnosed with cancer are therefore counseled for testicular tissue cryopreservation prior to the onset of cancer treatments (Avarbock et al. 1996). Fertility restoration using frozen testicular tissue (Avarbock et al. 1996; Keros et al. 2005; Onofre et al. 2020) has reported limited success in experimental studies (Yokonishi et al. 2014; Fayomi et al. 2019; Moussaoui et al. 2022; Whelan et al. 2022).

Cryopreservation of testicular tissue is technically challenging due to its complex architecture, which is comprised of an extracellular matrix, a heterogeneous mixture of germ cells, and somatic cells. Earlier studies have reported that the plasma membrane lipid composition differs among the cell populations of prepubertal and adult testes (Davis et al. 1966; Coniglio et al. 1975; Johnson and Pursel 1975; Lin et al. 2004). Therefore, the susceptibility of prepubertal and adult testicular cells to freeze–thaw-induced damage can vary due to the possible differences in the diffusion coefficient for penetrating cryoprotectants in testicular cells (Keros et al. 2005; Unni et al. 2012; Gironi et al. 2020). Further, the prepubertal testicular tissues are frozen with a cryopreservation medium designed for the adult testicular tissue. In adult testicular tissue the cell type of primary interest is spermatozoa, unlike in prepubertal tissue, where the retention of functional properties of all the cell lineages supporting spermatogenesis is more crucial.

Irrespective of cell type subjected to cryopreservation, the plasma membrane is believed to be the primary site for freeze–thaw-induced damage (Steponkus and Lynch 1989; Holt et al. 1992). Reactive oxygen species (ROS) generated by cryopreservation-induced oxidative stress predominantly target the polyunsaturated fatty acids (PUFAs) present in the plasma membrane (Tang et al. 2014), causing irreversible membrane changes. Further, freezing and thawing of cells can trigger the rearrangement of lipids (Bhojoo et al. 2018; Sun and Böckmann 2018), loss of cholesterol and phospholipids (Hinkovska-Galcheva et al. 1989; Buhr et al. 1994; Chakrabarty et al. 2007), and structural deformities in proteins (Bischof et al. 2002), which can collectively disrupt cellular function. Attempts have been made earlier to restore the function of cells subjected to the freeze–thaw process by exogenously supplementing membrane lipids (Odintsova et al. 2001, 2006; Odintsova and Boroda 2012) and free radical scavengers (Len et al. 2019; Liu et al. 2021). The presence of membrane lipids and antioxidants in the freezing medium is expected to improve the cryopreservation outcome by replenishing the lost membrane lipid components and preventing oxidative stress. In this study, we report the beneficial effect of membrane lipid and antioxidant-rich freezing medium on the outcome of prepubertal testicular tissue cryopreservation.

## Experimental procedures

### Testicular tissue collection, cryopreservation and thawing

**Fig. 1:**
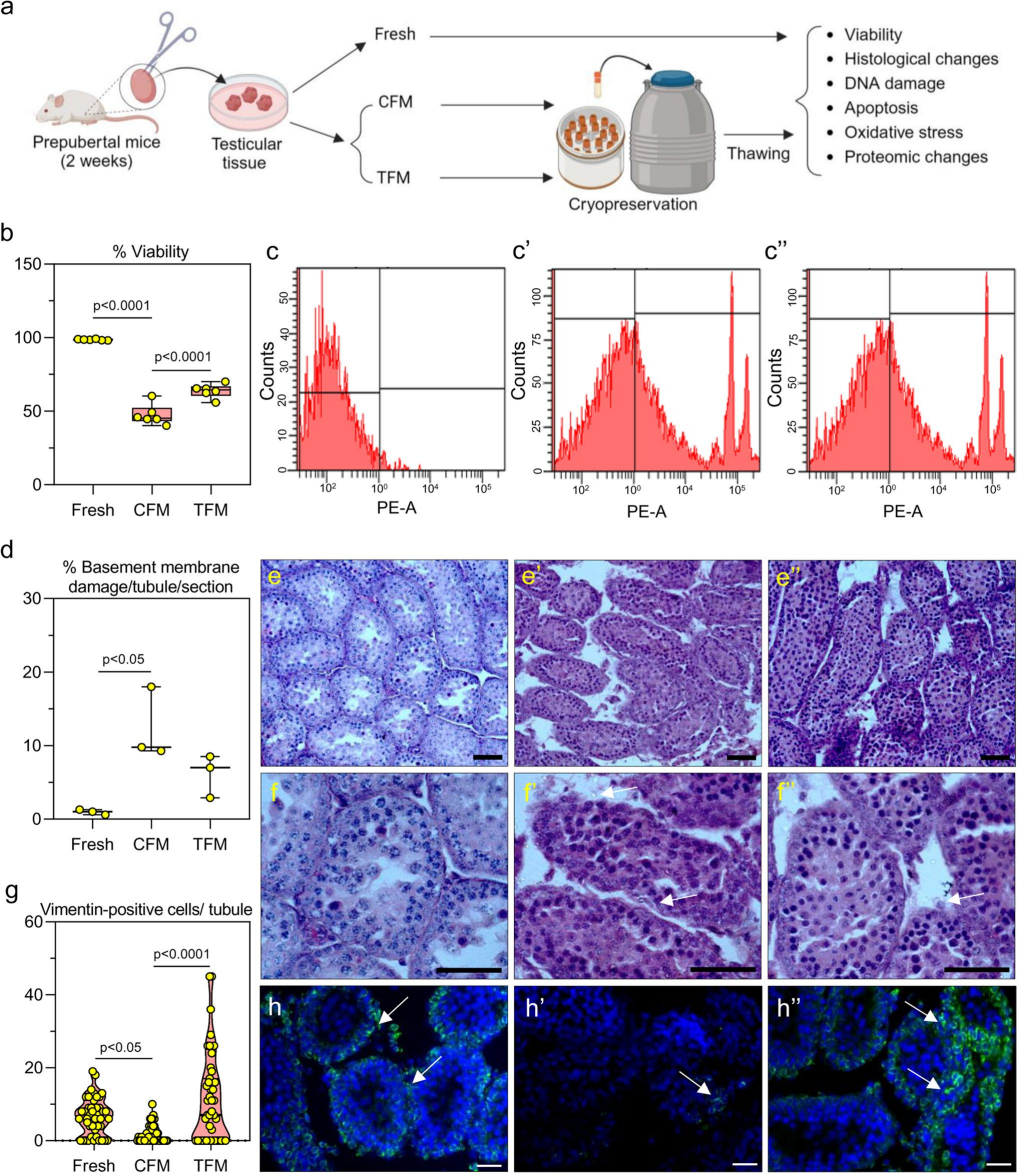
**a** Experimental design of mice prepubertal testicular tissue. The images were created with Biorender.com; **b** Effect of TFM on cell viability assessed by flow cytometry assay in prepubertal testicular tissue subjected to cryopreservation by slow freezing method (*N* = 6); Representative histogram for flow cytometry assessment of cells that are **c** unstained cells, from **c’** CFM and **c’’** TFM. The data is represented as Mean ± SEM; **d** Basement membrane damage assessed in prepubertal mice testicular tissue cryopreserved with CFM and TFM (*N* = 3); **e–f’’** Histopathological changes in prepubertal mice testicular tissue cryopreserved with CFM (e’ and f’ at 100 × and 400 × magnification, respectively) and TFM (e’’ and f’’ at 100 × and 400 × magnification, respectively). Arrows indicate basement membrane damage. Scale bar represents 100 μm; **g** Expression of vimentin in prepubertal testicular tissue after freeze–thaw process (*N* = 3). The data is represented as Mean ± SEM; Representative images of IHC showing vimentin expression in prepubertal testicular tissue cryopreserved with **h’** CFM and **h’’** TFM. White arrowheads indicate cells positive for vimentin. The scale bar represents 20 μm

Testicular tissues were collected from inbred prepubertal Swiss albino male mice (2 weeks) maintained at the Central Animal Research Facility, Manipal Academy of Higher Education, Manipal, India. Prepubertal mouse testicular tissue was cryopreserved by slow freezing protocol, as previously described (Onofre et al. 2020), with minor modifications. Briefly, the testicular tissues were collected in DMEM/F12 (11320033, Gibco, USA) medium on ice, after euthanizing the mice by cervical dislocation. Immediately after collection, the testis was decapsulated and cut into 3 mm^2^ pieces. The testicular tissue was then transferred to a cryovial (P60116, Abdos, USA) containing 500 μL of either control freezing medium (CFM) composed of 5% dimethyl sulfoxide (DMSO, D4540, Sigma, USA) and 30% fetal bovine serum (FBS, CCS-500-SA-U, Genetix Biotech, Cell clone™, India) in DMEM/F12 medium or in the test freezing medium (TFM) composed of 2.5 mg/mL soy lecithin (P5638, Sigma Aldrich, USA), 1.0 mg/mL phosphatidylethanolamine (P1223, Sigma Aldrich, USA), 0.25 mg/mL phosphatidylserine (P7769, Sigma Aldrich, USA), 1.0 mg/mL cholesterol (10367201001730, Merck, India), 0.004 mg/mL sodium selenite (214485, Sigma Aldrich, USA), 0.6 mg/mL vitamin C (A4544, Sigma Aldrich, USA), 5% DMSO and 30% FBS in DMEM/F12 medium, which was formulated based on our pilot study (Supplementary Table 1). The cryovials were then placed in an isopropanol chamber (Mr. Frosty™ Freezing Container, 5100–0001, Thermofisher Scientific, USA) at -80 °C for 24 h and then stored in liquid nitrogen for a minimum of one week (Fig. 1a).

Thawing of testicular tissue was performed according to the protocol described by Milazzo et al. (2008) with minor modifications. Briefly, the samples were thawed rapidly by placing the cryovials in a water bath maintained at 37 °C for 2 min. Tissues were then placed sequentially in thawing solution 1 (TS1; 2.5% DMSO, 0.05 M sucrose and 10% FBS in DMEM/F12 medium) followed by thawing solution 2 (TS2; 1% DMSO, 0.05 M sucrose and 10% FBS in DMEM/F12 medium), thawing solution 3 (TS3; 0.05 M sucrose in DMEM/F12 medium) and finally in thawing solution 4 (TS4; DMEM/F12 Medium) for 5 min each at room temperature. The tissues were kept on ice until further handling.

### Collection of testicular cells by enzymatic digestion

Isolation of prepubertal testicular cells was performed as described earlier (Nayak et al. 2016). Briefly, the testicular tissue was suspended in DMEM/F12 media containing 1.0 mg/mL trypsin (49041, Gibco, USA) and collagenase type IV (17104–019, Gibco, USA) at 37 °C for 30 min in a shaking water bath. DMEM/F12 containing 10% FBS was used to neutralize trypsin action, and the cells were filtered using a 70 μm (22–363-548, Fisher Scientific, USA), followed by a 40 μm cell strainer (22–363-547, Fisher Scientific, USA). The flow through was then centrifuged at 100 × g for 10 min. The obtained cell pellet was re-suspended in phosphate-buffered saline (PBS) and placed on ice until further assessment.

### Assessment of viability in testicular cells

Immediately after cell isolation from the tissue, the number of viable cells was determined by the flow cytometric method. Briefly, 1.0 × 10^6^ cells obtained from each condition were resuspended in Ca^2+^/Mg^2+^ free PBS, centrifuged at 150 × g at room temperature for 5 min and the wash step was repeated. The cell pellet was gently resuspended in 1 mL of serum-free basal medium. Before acquiring the samples on the flow cytometer, propidium iodide (PI) was immediately added to the tubes at a final concentration of 50 µg/mL. The sample was gently mixed and acquired on a BD LSRII analyzer (33300478, USA). An unstained tube of cells was used as a negative control. Analysis was performed using BD FACSDiva™ Software. The percentage of cells negative for PI was considered viable.

### Histology and immunohistochemistry

To assess the histological changes after the freeze–thaw process, testicular fragments were first fixed for 48 h in Bouin’s fixative, followed by storage in 70% ethanol. The tissues were then successively dehydrated using graded alcohol before embedding in paraffin. Five μm thick sections were prepared, stained with hematoxylin and eosin, and observed under a light microscope at 400 × magnification.

Immunohistochemical staining was performed by deparaffinization of the slides on a dry bath maintained at 60 °C, followed by placing the slides in xylene for 15 min. The slides were then serially rehydrated in 100, 95, and 70% ethanol for 2 min each. Next, the slides were placed in 0.01 M sodium citrate buffer (pH 6.0) and placed in a boiling water bath for antigen retrieval for 20 min. Permeabilization was performed with 0.3% Triton X-100 for 15 min and blocked with 1% bovine serum albumin (BSA) for 1 h at 37 °C. The slides were treated with anti-vimentin primary antibody (1:200; 5741, Cell Signaling Technology, USA) overnight. After washing in PBS thrice, the sections were incubated with goat anti-rabbit Alexa fluor™ 488 secondary antibody for 1 h at 37 °C. Excess and non-specific binding was removed by thoroughly washing the slides with PBS. The sections were then mounted using Fluoroshield™ with DAPI (F5932, Sigma-Aldrich, USA) and imaged using QCapture Pro 7 software, USA, equipped with a fluorescence microscope.

### Estimation of malondialdehyde (MDA), glutathione (GSH), and protein carbonyl content (PCO)

Testicular tissue homogenate was prepared in PBS. After estimating the protein level by Bradford’s method (Ernst and Zor 2010), the oxidative stress generated during the freeze–thaw process was evaluated by measuring the MDA, GSH, and PCO content in the testicular homogenate as described earlier (Dcunha et al. 2023).

### Isolation of SGCs, leydig cells, and sertoli cells from prepubertal testicular tissue

Germ cells, Leydig cells, and Sertoli cells from testicular tissue were isolated using the method described previously (Chang et al. 2011). Briefly, the testicular tissue pieces were digested with 0.5 mg/mL of collagenase for 15 min in a 37 °C shaking water bath, after which the suspension was layered on a 5% Percoll™ Plus (17–5445-02, GE Healthcare Bio-Science, Sweden) gradient in Hanks balanced salt solution (HBSS) and allowed to settle for 20 min at room temperature, during which the seminiferous tubules settled at the bottom of the tube. The Leydig cell-rich top layer was collected and centrifuged at 100 × g for 10 min. The tubules that settled at the bottom of the gradient were collected and further subjected to digestion in 1.0 mg/mL of trypsin for 15 min at 37 °C in a shaking water bath. Immediately, DMEM/F12 media with 10% FBS was added, and the cell suspension was sequentially passed through a cell strainer of pore size 70 and 40 μm. The filtrate containing the cells was centrifuged at 100 × g for 10 min to obtain the germ cell fraction. Subsequently, the Sertoli cells were collected by inverting the 40 μm cell strainer and washed repeatedly with DMEM/F12 media to flush the cells followed by centrifugation at 100 × g for 10 min.

### Immunofluorescence

The cell suspension was air-dried on a clean glass slide, after which they were fixed with 4% paraformaldehyde (PFA) overnight at 4 °C. The cells were subsequently permeabilized for 10 min with 0.3% Triton X-100 in PBS. The cells were rinsed in PBS, then blocked for 30 min with PBS containing 1% BSA and 0.1% tween-20, and then treated overnight with primary antibodies: Anti-phospho histone H_2_AX (1:250; 9718S, Cell Signaling Technology, USA), Annexin V (1: 200; 8555S, Cell Signaling Technology, USA), 3β-hydroxysteroid dehydrogenase (1:25; Sc-37392, Santa Cruz Biotechnology, USA), VASA (1: 200; Sc-48705, Santa Cruz, USA) and SOX9 (1:300; PA5-81966, Thermo Fisher Scientific, USA) in PBST at 4 °C. The cells were mounted using Fluoroshield™ with DAPI after being treated with the appropriate secondary FITC-antibody for 1 h at 37 °C. Cells were visualized under a fluorescence microscope and the images were captured using QCapture Pro 7 software, USA. A minimum of 500 cells were counted under 400 × magnification, and the data was reported as a percentage of the cells with positive signals for the protein of interest.

### Gene expression analysis

Gene transcription analysis for Tumor protein P53 (*p53*), Apoptosis regulator BAX (*Bax*), B-cell lymphoma 2 (*Bcl-2*), Cytochrome C (*Cyt c*), Caspase-3 (*Casp3*), Superoxide dismutase (*Sod1*), Glutathione peroxidase 4 (*Gpx4*) and Catalase (*Cat*) was performed using a real-time PCR. RNA isolation (TRI reagent®, T9424, Sigma Aldrich, USA), cDNA synthesis, and qPCR analysis were performed as specified earlier (Kumari et al. 2021). A detailed list of primers used in the study is provided in Supplementary Table 2.

### Proteomic analysis of SGCs from prepubertal testicular tissue

Protein extraction from SGCs and processing (reduction, alkylation, acetone precipitation, and enzymatic digestion) for tandem mass tag (TMT) labeling was performed using methods described earlier (Najar et al. 2021). The samples from each condition were labeled using TMT 10plex™ (Thermo Scientific, Bremen, Germany) reagents. The peptides from the fresh group were labeled with 126, the CFM with 127N, and TFM with 127C. The labeled peptide samples were then incubated for 1 h at room temperature and the reaction was quenched with hydroxylamine. Before pooling the samples, equal amounts of peptides were taken from each sample for the TMT label check. Equal amounts of labeled peptides were then pooled and vacuum-dried for further processing.

Peptide fractionation and C18 clean-up were performed as described earlier (Karthikkeyan et al. 2020). For LC–MS/MS data acquisition, Easy-nLC™1200 (Thermo Scientific, Odense, Denmark) coupled with Orbitrap Fusion Tribrid mass spectrometer (Thermo Fisher Scientific, Bremen, Germany) was used. The fractionated and dried peptide samples were resuspended in 0.1% formic acid and loaded onto a 2 cm trap column (nanoViper, 3 µm C18 Aq) (Thermo Fisher Scientific). Peptide separation was performed using a 15 cm analytical column (nanoViper, 75 µm silica capillary, 2 µm C18 Aq) at 300 nL/min flow rate. The solvent gradients were set as a linear gradient of 5–35% solvent B (80% acetonitrile in 0.1% formic acid) for 90 min with a total run time of 120 min.

The data was acquired in data-dependent mode, where positively charged ions (peptide precursors) ranging between 400–1600 m/z were filtered using quadrupole and trapped in C-trap until AGC reaches 2e^5^ for 50 ms, followed by ion detection in Orbitrap under the resolution of 120,000 at 200 m/z. The peptides with charge states of 2–7 were considered for analysis, and the dynamic exclusion rate was set as 45 s with a mass tolerance of 10 ppm. For the MS/MS level, a higher collision energy dissociation (HCD) fragmentation mode with a normalized collision energy of 35% was used at a resolution of 60,000 at 200 m/z using the Orbitrap mass analyzer with a maximum injection time of 200 ms. Data acquisition was carried out in technical triplicates for each fraction.

The MS/MS raw files were searched for peptides and proteins using Mascot and Sequest HT search engines in Proteome Discoverer v2.2 (Thermo Scientific). The proteomic database search was performed against *Mus musculus* reference proteome (version: 109) with known mass spectrometry contaminants. The protein sequences were theoretically digested using trypsin protease enzyme with a maximum of one missed cleavage and searched against the LC–MS/MS generated data with 10 ppm and 0.02 Da precursor and fragment mass tolerance, respectively. The common modifications included in the search are methionine oxidation and protein N-terminal acetylation as variable modifications; Carbamidomethylation at cysteine and TMT 6plex as fixed modification. The minimum precursor mass was set as 350 Da and the maximum precursor mass as 5000 Da. A maximum false discovery rate (FDR) of 1% was set separately at protein, peptide, and PSM levels.

### Bioinformatics and statistical analysis

All the numerical data are expressed as the mean and standard error of mean (Mean ± SEM) and, were analyzed using GraphPad Prism 8.0 (GraphPad Software Inc., USA). The statistical significance of differences among groups was determined by one-way ANOVA followed by Tukey’s multiple comparison test. For grouped analysis, two-way ANOVA was performed followed by Tukey’s multiple comparison test. A *p*-value ≤ 0.05 indicated statistical significance. For mass spectrometric analysis, the data acquired after Proteome Discoverer analysis were used for further downstream bioinformatics data analysis to identify the differentially expressed proteins (DEPs). The abundance values corresponding to each protein were subjected to median normalization, only the abundance values present in two out of three replicates were considered. Further, the normalized abundance values were taken for the fold change calculation, comparing CFM versus fresh conditions and TFM-treated sample versus CFM followed by the *p*-value calculation using Student’s t-test. A fold change cut-off of 1.5 was set for DEPs and the proteins with *p*-value ≤ 0.05 were considered as significant. The DEPs were subjected to further functional enrichment analysis. The pathway analysis was carried out using Reactome (https://reactome.org) and gene ontology (GO) analysis using gprofiler (https://biit.cs.ut.ee). The protein–protein interaction network analysis was carried out using the search tool for the retrieval of interacting genes/ proteins (STRING) database and Cytoscape version 3.9.1 (Shannon et al. 2003).

## Results

### Cryopreservation of prepubertal testicular tissue in TFM improves the survival of testicular cells

The flow cytometric data revealed that the freeze–thaw process resulted in a significant decrease (*p* < 0.0001) in the number of viable cells when cryopreserved using CFM. However, cryopreservation using TFM resulted in a significantly higher (*p* < 0.001) percentage of viable cells, compared to CFM (Fig. 1b, c–c’’, and Supplementary Fig. 1). Further, we assessed the freezing point of the TFM to understand whether the improvement in cryosurvival of prepubertal testicular cells is mediated through any alteration in the freezing point. However, the freezing point of CFM and TFM were almost similar suggesting that the additional components in the TFM did not alter the freezing point (Supplementary Fig. 2). To confirm the uptake of the membrane lipids by testicular cells from the TFM during the freeze–thaw process, instead of cholesterol we used NBD-cholesterol in TFM. The presence of NBD-cholesterol in the frozen-thawed testicular cells indicated the uptake of membrane lipids by the testicular cells during cryopreservation (Supplementary Fig. 3).

### Histological changes in prepubertal testicular tissue subjected to cryopreservation

Histological analysis suggested considerable changes in the testicular tissue architecture following the freeze–thaw process. The tissues frozen in CFM exhibited detachment of cells from the basement membrane (Fig. 1d) and higher intertubular space than the fresh tissue and TFM (Fig. 1e-f’’). Cells expressing vimentin, a marker used to study cell-to-cell integrity, were significantly lower (*p* < 0.05) in tissue cryopreserved in CFM compared to the fresh tissue. However, a twofold higher (*p* < 0.0001) percentage of vimentin-positive cells was observed in the tissue frozen in the TFM compared to CFM (Fig. 1g-h’’).

### Cryopreservation of prepubertal testicular tissue in TFM minimizes the freeze–thaw-induced DNA damage, and apoptosis in testicular cells by mitigating oxidative stress

**Fig. 2:**
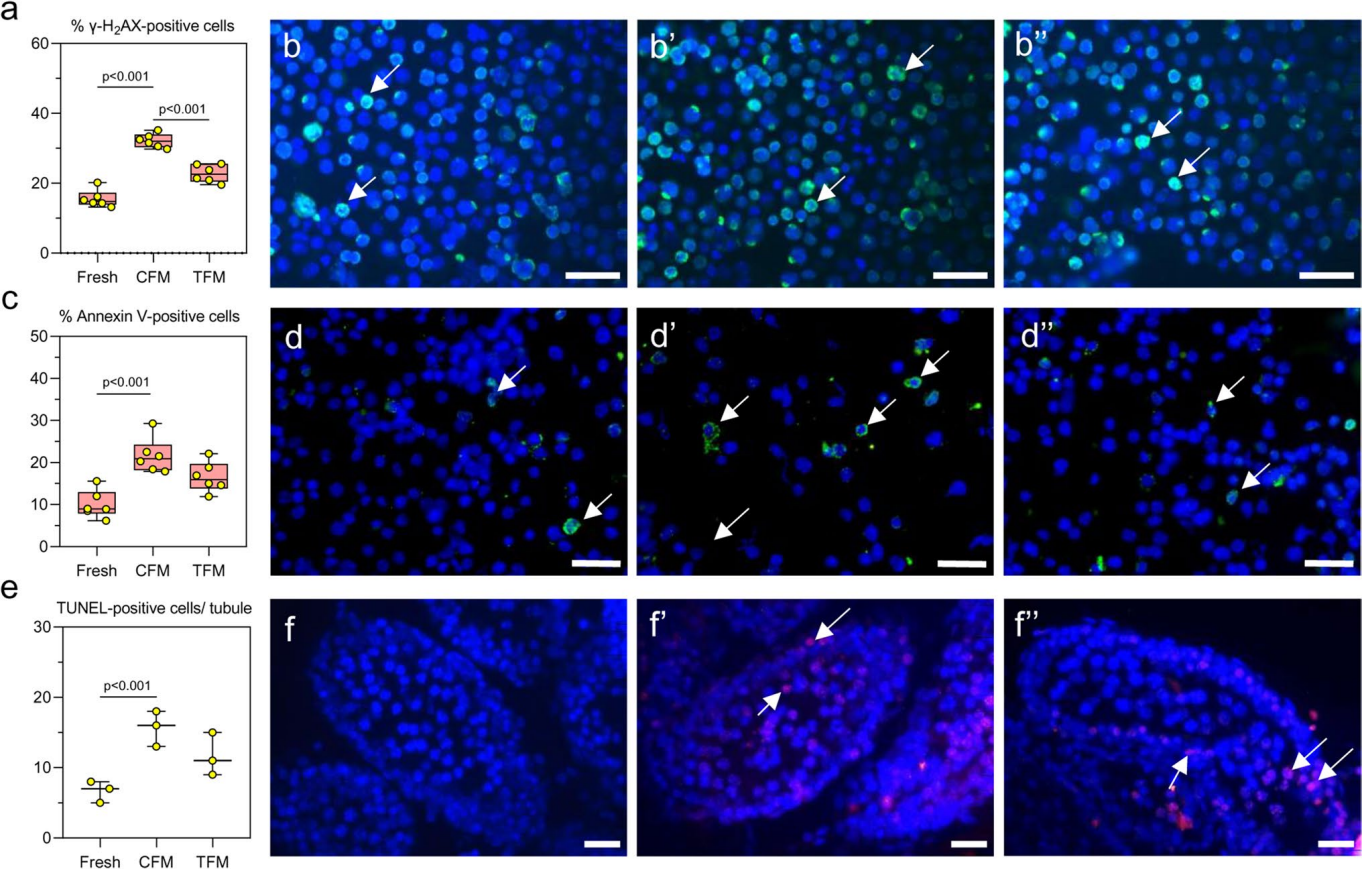
Assessment of DNA integrity, and apoptosis in prepubertal mice testicular tissue cryopreserved with CFM and TFM. **a** Assessment of DNA damage by γ-H_2_AX expression in prepubertal testicular tissue (*N* = 6); Representative images showing γ-H_2_AX positive cells (400x) in **b** Fresh, **b’** CFM and **b’’** TFM group. White arrowheads indicate γ-H_2_AX positive cells; the Scale bar represents 20 μm. **c** Annexin V expression (*N* = 6). The data is represented as Mean ± SEM; Representative images showing testicular cells positive for Annexin V from **d** Fresh, **d’** CFM and **d’’** TFM group; **e** Effect of TFM on DNA damage assessed by TUNEL assay (*N* = 3); The data is represented as Mean ± SEM; Representative images showing TUNEL-positive cells in **f** Fresh tissue and prepubertal mice testicular tissue subjected to freeze–thaw process using **f’** CFM and **f’’** TFM. White arrowheads indicate TUNEL-positive cells; Scale bar represents 20 μm

**Fig. 3:**
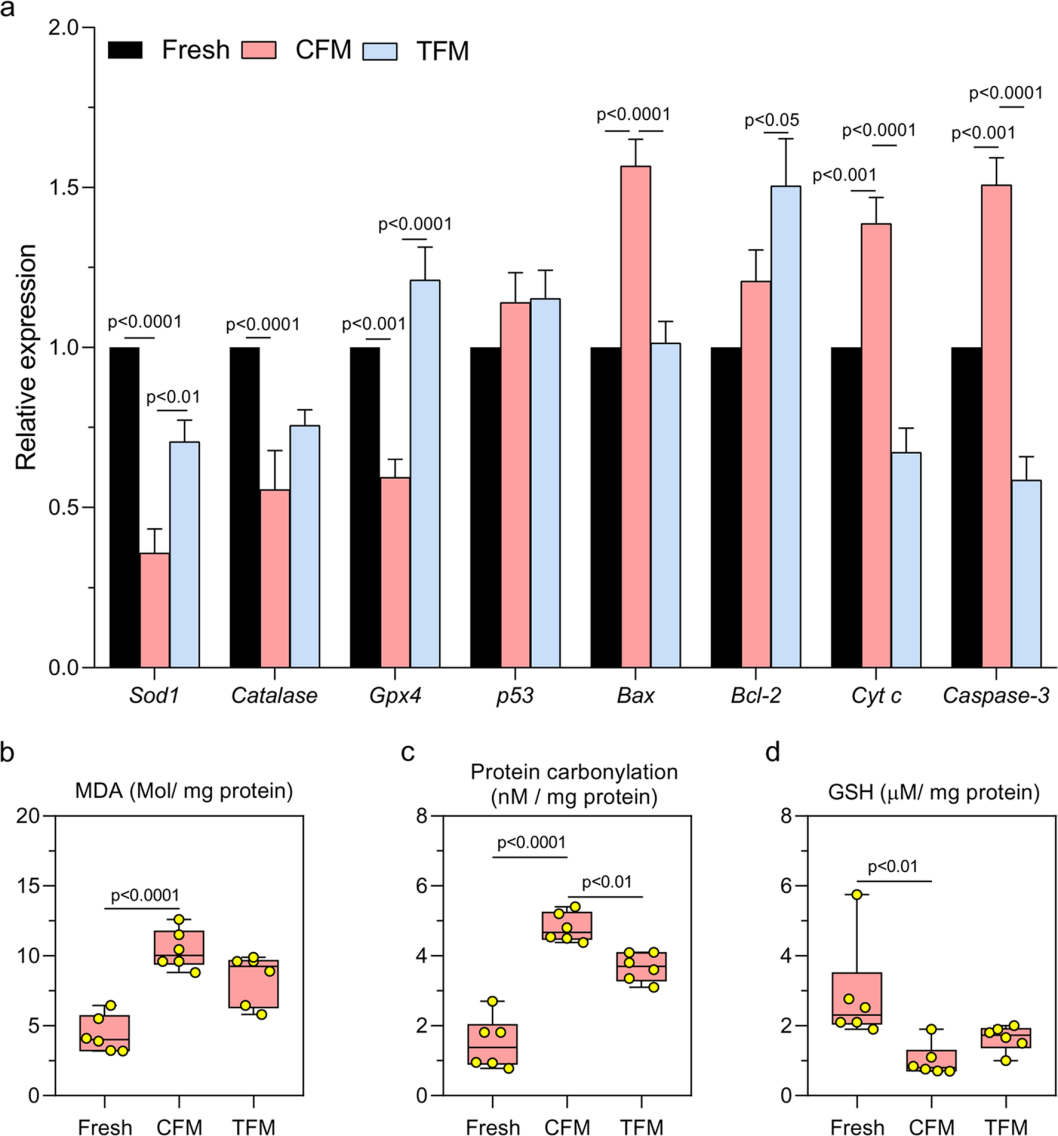
**a** Gene expression pattern for *Gpx4*, *Sod1*, *Cat*, *p53*, *Bcl-2*, *Bax*, *Cytc,* and *Casp3* analyzed by qRT-PCR in prepubertal mice testicular tissue cryopreserved with CFM and TFM (*N* = 6). The data is represented as Mean ± SEM; Assessment of **b** Malondialdehyde; **c** Protein carbonyl content; **d** Glutathione level in prepubertal mice testicular tissue cryopreserved with CFM and TFM (*N* = 6). The data is represented as Mean ± SEM

A significant increase (*p* < 0.0001) in the number of testicular cells with DNA double-strand breaks (γ-H_2_AX positive cells) was observed in tissues cryopreserved in CFM compared to the fresh tissue. In comparison, a significantly lower (*p* < 0.0001) percentage of γ-H_2_AX positive cells was observed compared to those frozen in CFM (Fig. 2a-b’’). Similar observations were made with adult testicular tissue (Supplementary Fig. 4a-c). Similarly, in testicular tissues frozen in TFM, Annexin-V positive cells were lower than (non-significant) those cryopreserved in CFM (Fig. 2c-d’’). TUNEL assay was performed in tissue sections to understand the extent of DNA damage induced by the freeze–thaw process. A significant increase (*p* < 0.01) in the number of TUNEL-positive cells per tubule was observed in tissue frozen with CFM compared to the fresh tissue, which was lower in tissue cryopreserved in the TFM (Fig. 2e-f’’). However, the difference was statistically not significant. These results were further confirmed by assessing the mRNA expression of genes involved in the apoptosis. Expression of *p53* did not differ between the CFM and TFM groups. However, the expression of the *Bax* (*p* < 0.0001), *Cyt C* (*p* < 0.0001), and *Capase-3* (*p* < 0.0001) was decreased, while the anti-apoptotic gene *Bcl-2* showed an increased expression (*p* < 0.05) in TFM compared to the tissues frozen in CFM (Fig. 3a). Further, a significant increase in the expression of *Gpx4* (*p* < 0.0001) and *Sod1* (*p* < 0.01), and a non-significant increase in *Catalase* in the TFM group was observed compared to CFM. Compared to fresh tissue, a significant increase (*p* < 0.0001) in MDA level (Fig. 3b) and PCO (Fig. 3c) content was observed in tissues cryopreserved in CFM. Further, the GSH level was significantly lower (*p* < 0.01) in tissues frozen in CFM compared to fresh tissue (Fig. 3d). On the other hand, a significant decrease in PCO content (*p* < 0.01), and a non-significant decrease in MDA and GSH level was observed in tissues frozen with TFM.

### TFM improves survival and reduces apoptosis in germ cells

**Fig. 4:**
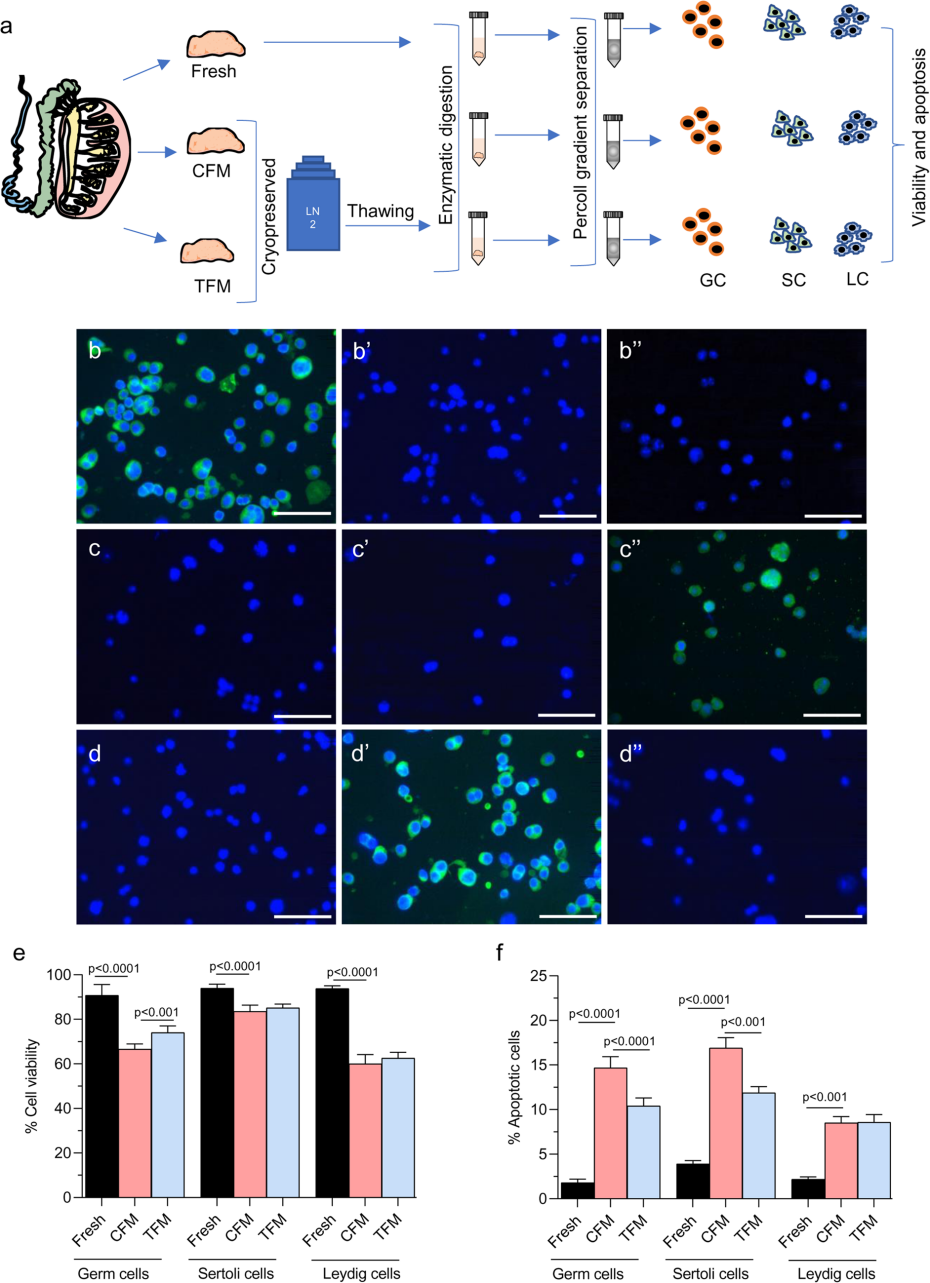
Effect of freeze–thaw process on the survival of germ, Sertoli, and Leydig cells of prepubertal mice testicular tissue. **a** Experimental outline for isolation of various testicular cells; Representative images to depict characterization of germ cells by studying VASA expression- **b** Germ cells, **b’** Leydig cells, and **b’’** Sertoli cells; Representative images to depict characterization of Sertoli cells by studying SOX9 expression- **c** Germ cells, **c’** Leydig cells, and **c’’** Sertoli cells; Representative images to depict characterization of germ cells by studying β-HSD expression- **d** Germ cells, **d’** Leydig cells, and **d’’** Sertoli cells; **e** Assessment of viability by trypan blue dye exclusion method (*N* = 6); **f** Apoptosis in isolated germ, Sertoli, and Leydig cell fraction isolated from prepubertal mice testicular tissue assessed by Annexin-V staining (*N* = 6). The data is represented as Mean ± SEM

The isolated germ, Leydig, and Sertoli cells were characterized using specific markers by immunofluorescence (Fig. 4a-d’’). The freeze–thaw process of testicular tissue resulted in a significant reduction in viability of all three cell types (*p* < 0.001) (Fig. 4e). However, in the tissues frozen using TFM, a significantly higher percentage of survival was observed in the germ cell fraction compared to those frozen with CFM. There was no change in the Leydig and Sertoli cell survival compared to the CFM. In addition, a significantly (*p* < 0.0001) higher percentage of apoptotic cells (Annexin-V positive cells) were observed in Leydig, germ, and Sertoli cells when frozen with CFM compared to the fresh tissue. Comparatively, in the germ cells and Sertoli cells, a significant decrease (*p* < 0.0001) in Annexin-V positive cells was observed in tissue cryopreserved using the TFM. However, it did not differ in the Leydig cell fraction compared to CFM (Fig. 4f).

### Proteomic changes in prepubertal germ cells cryopreserved using CFM and TFM

**Fig. 5:**
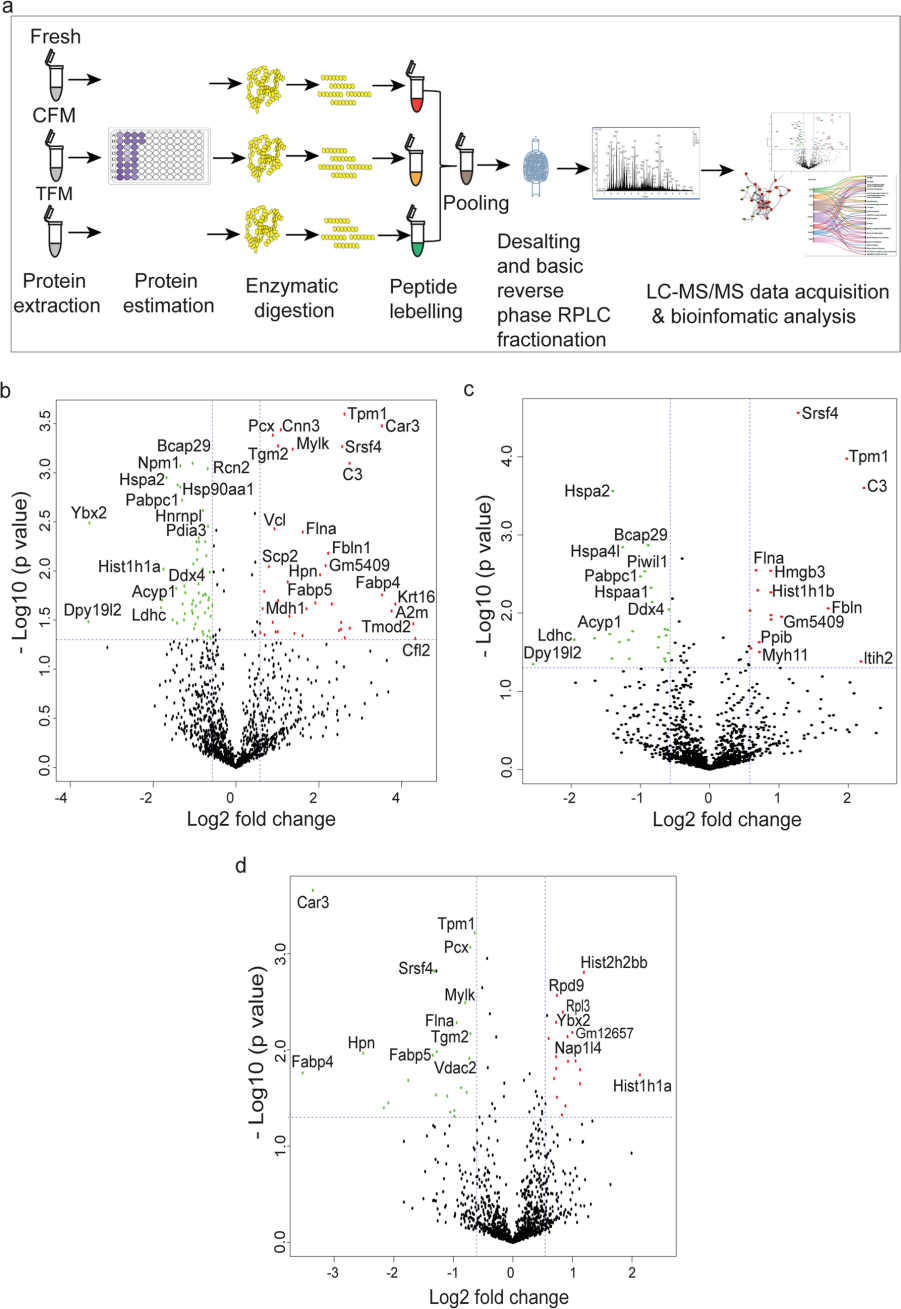
Proteomic alterations in spermatogonial germ cells (SGCs) isolated from prepubertal mice testicular tissue cryopreserved with CFM and TFM. **a** Experimental outline; **b** Volcano plot depicting significantly up- and downregulated proteins in spermatogonial germ cells from tissue cryopreserved in CFM compared to fresh tissue; **c** Volcano plot depicting significantly up- and downregulated proteins in spermatogonial germ cells from tissue cryopreserved in TFM compared to fresh tissue; **d** Volcano plot depicting significantly up- and downregulated proteins in spermatogonial germ cells from tissue cryopreserved in CFM compared to TFM; Red color indicates significantly upregulated and green color indicates significantly downregulated proteins

To understand the changes in the protein expression profile of prepubertal SGCs subjected to the freeze–thaw process, we performed a TMT-based quantitative proteomics approach (Fig. 5a). The freeze–thaw process induced a significant change in the protein profile of the SGCs in cryopreserved tissues both in CFM and TFM. A total of 1401 proteins were detected from the proteomic analysis, at a false discovery rate of 1%. Among the proteins detected, Spr, Samm50, Fah, Eif1, Sars, Ralb, F2, Mfsd7c, Flvcr2, Comp, F5, H2ke6, A2m, Set, Ssb, 1700037H04Rik, and Msn have not been reported in the mouse SGCs so far. Further, in 1401 proteins identified, 97 proteins were differentially expressed in the SGCs of tissues frozen with the CFM group compared to fresh tissue. Comparatively, 40 proteins were differentially expressed in SGCs of testicular tissues frozen with TFM compared to CFM. To validate the proteomics data, the differential expression of DDX4 (VASA), one of the markers for SGCs was assessed by immunofluorescence in SGCs (Supplementary Fig. 5). The VASA-positive SGCs were observed to be higher (non-significant) in the TFM group compared to CFM.

The volcano plot depicts significantly overexpressed (above 1.3-fold) and downregulated proteins (< 0.7-fold) in SGCs cryopreserved with CFM (Fig. 5b) and TFM (Fig. 5c) compared to the fresh tissue and, TFM (Fig. 5d) in comparison to the CFM. In comparison to the SGCs of the fresh testicular tissue, Cfl2, Tmod2, Krt16, A2m, Fabp4, Car3, ltih2, C3, Serpinc1, and tpm1 were the top 10 upregulated proteins in SGCs of CFM group. While Dpy19l2, Ybx2, Ldhc, Acyp1, Hist1h1a, Hspa2, Nasp, Nap1l4, Sccpdh and Hspa4l were the top 10 significantly downregulated proteins. However, in case of SGCs from TFM, Hist1h1a, Hist2h2bb, Fkbp6, Rps20, Hist1h1c, Gm12657, Npm1, Nap1l4, hist4h4 and Rpl3 proteins were found to be overexpressed, while Tpm1, Tgm2, Pcx, Vdac2, Ldhal6b, Mylk, Cox2, Flna, Tpm2, and Myh11 were significantly downregulated. Overall, SGCs from the CFM group exhibited 39 significantly upregulated and 58 significantly downregulated proteins compared to SGCs from fresh tissue. Similarly, in SGCs from the TFM group, 18 proteins were overexpressed while 22 proteins were found to be significantly downregulated. Among the top 10 DEPs in the SGCs of frozen-thawed samples, cfl2, Mylk, Flna, and Myh11 are reported to be membranous proteins. Krt16, Fabp4, Car3, Ltih2, Tpm1, Ybx2, Ldhc, Acyp1, Hspa2, Sccpdh, Hspa4l, Fkbp6, Rps20, Tgm2, Pcx, Vdac2, Ldhal6b, Cox2 and Pdhb were cytoplasmic, and tmod2, Hist1h1a, Nasp, Nap1l4, Npm1, Hist2h2bb, Hist1h1c and Hist4h4 in the nucleoplasm.

**Fig. 6:**
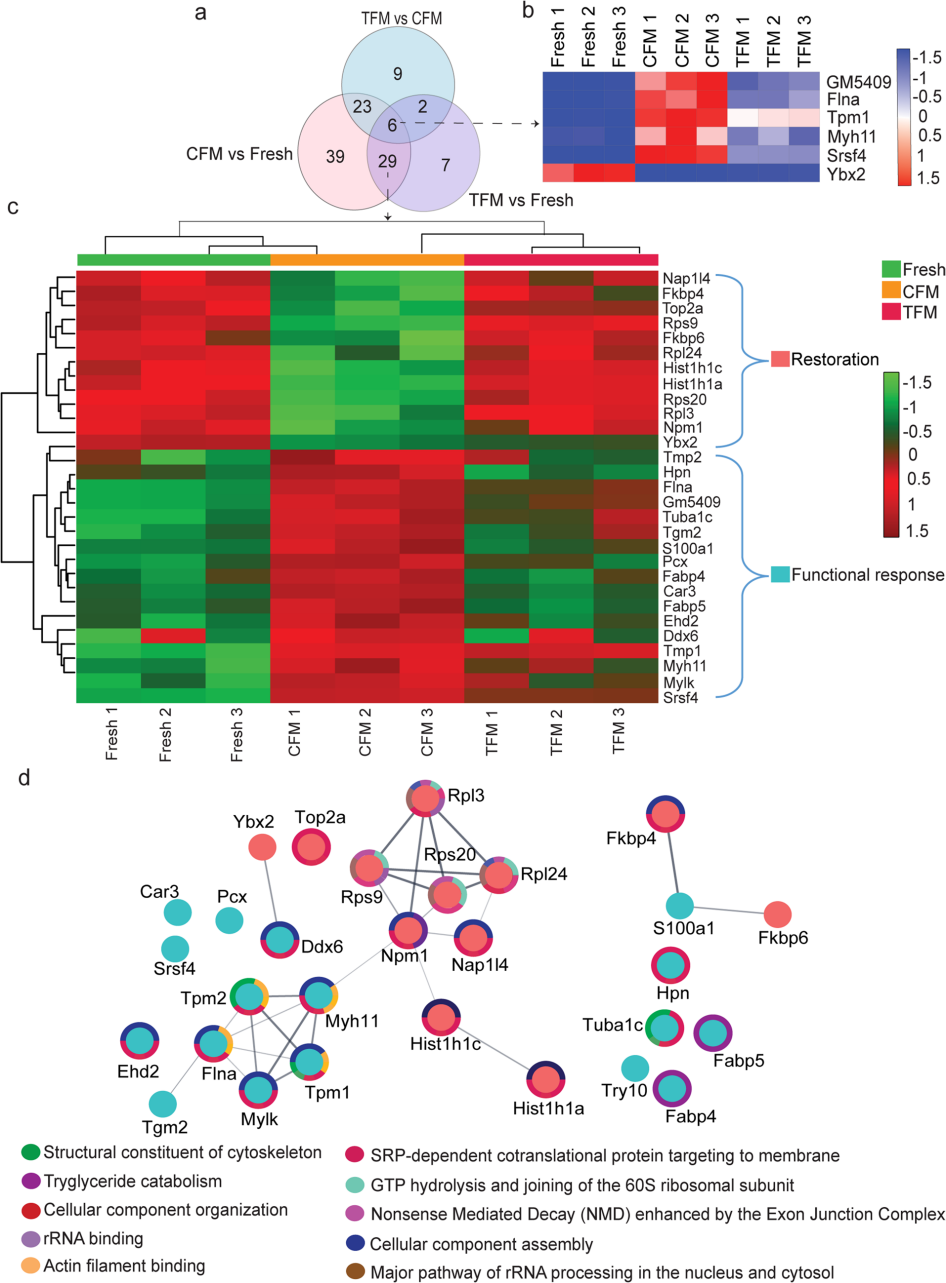
The altered protein expression in spermatogonial germ cells isolated from prepubertal mice testicular tissue cryopreserved with CFM and TFM. **a** Venn diagram showing proteins that are common and uniquely expressed in the three experimental conditions; **b** Differential expression of proteins from spermatogonial germ cells isolated from fresh, CFM, and TFM **c** Differential expression of proteins from spermatogonial germ cells isolated from CFM, and TFM; **d** Interactome analysis of the common proteins expressed in spermatogonial germ cells from fresh, CFM, and TFM; Node color indicates the protein expression and edge color indicates the various biological processes they are involved in

A heat map showing all the DEPs is represented in Supplementary Fig. 6. Among the DEPs, 6 proteins (Gm5409, Flna, TPM1, Myh11, Srsf4, and Ybh2) were observed to be common for fresh, CFM and TFM groups (Fig. 6a, b). Further, 29 proteins were differentially expressed and were common to CFM and TFM groups (Fig. 6a, c). The protein–protein interaction study demonstrated that 12 proteins, downregulated in TFM, similar to the fresh tissue, were involved in cell restoration after the freeze–thaw process (Fig. 6d, node color red). Further, 17 proteins were upregulated compared to CFM, which showed similar expression patterns with the fresh group and demonstrated to be involved in cell function restoration.

**Fig. 7:**
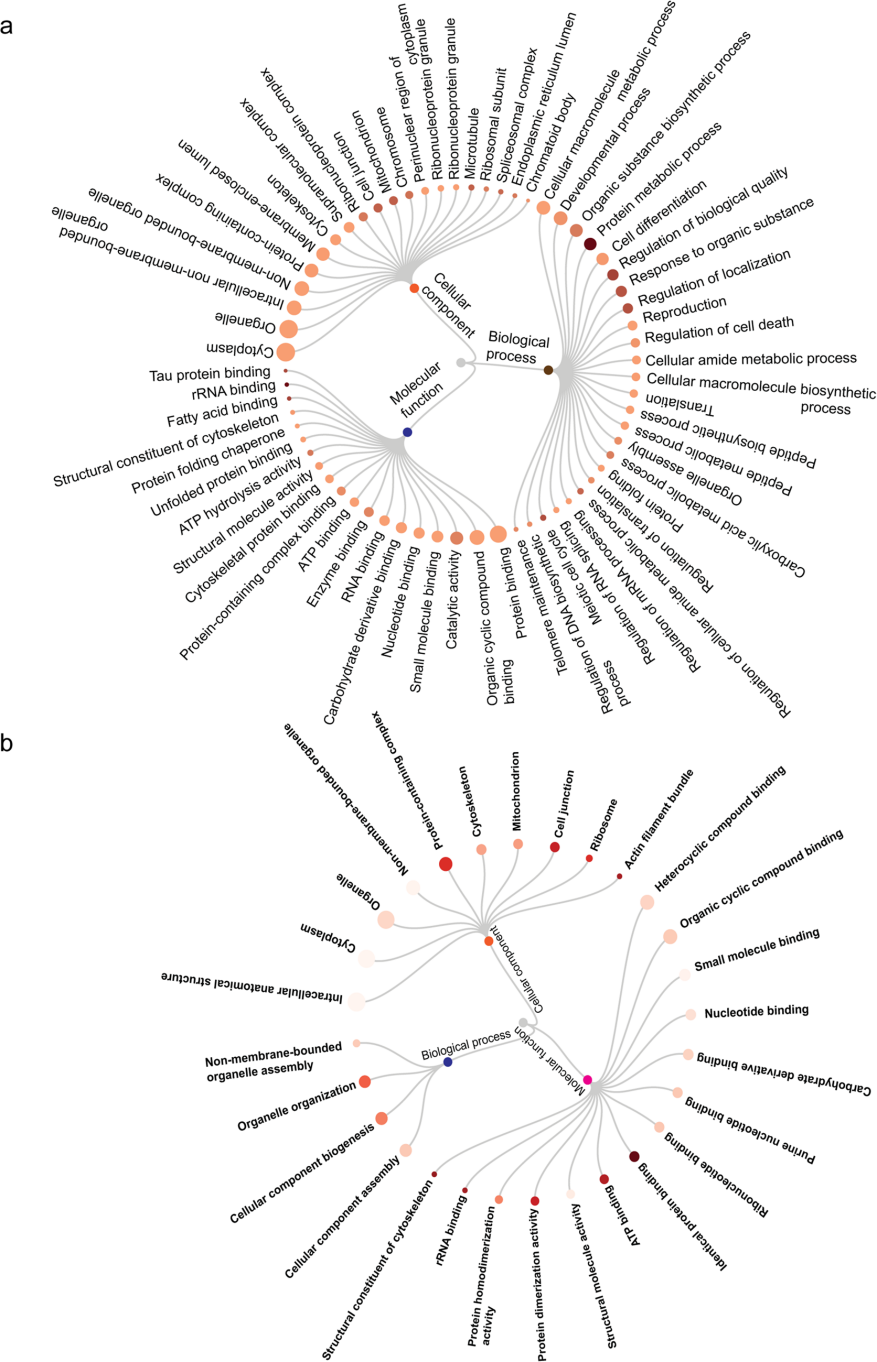
Gene ontology analysis of the significantly up- and downregulated proteins in spermatogonial germ cells from tissue cryopreserved in **a** CFM compared to fresh tissue; **b** CFM compared to TFM

To comprehend the changes in the SGCs during the freeze–thaw process, GO analysis was performed. The data suggests that the majority of the DEPs function in processes such as cellular macromolecule metabolic process, developmental process, organic substance biosynthesis process, protein metabolic process, cell differentiation, regulation of biological quantity, response to organic substance, regulation of localization, reproduction, regulation of cell death (Fig. 7a, biological process). The molecular function revealed that the DEPs are involved in protein binding, organic cyclic compound binding, catalytic activity, small molecule binding, nucleotide binding, carbohydrate derivative binding, and enzyme binding (Fig. 7a, molecular function). Based on the cellular component analysis, most proteins are present in the cytoplasm, organelle, intracellular non-membrane-bounded organelle, non-membrane-bounded organelle, protein-containing complex, membrane-enclosed lumen, cytoskeleton, supramolecular complex, ribonucleoprotein complex (Fig. 7a, cellular component). Compared to the CFM, freeze–thaw-induced insult was less in spermatogonial cells obtained from the tissue cryopreserved using the TFM (Fig. 7b). GO analysis of the DEPs of tissues cryopreserved with TFM suggested involvement in cellular component assembly, cellular component biogenesis, organelle organization, and non-membrane bound organelle assembly (Fig. 7b, biological process). The molecular function revealed that proteins are involved in heterocyclic compound binding, organic cyclic compound binding, small molecule binding, nucleotide binding, carbohydrate derivative binding, and purine nucleotide binding (Fig. 7b, molecular function). Cellular component analysis revealed that the proteins are present in intracellular anatomical structure, cytoplasm, organelle, non-membrane-bounded organelle, protein-containing complex, cytoskeleton, mitochondrion, cell junction, ribosome, and actin filament bundle (Fig. 7b, cellular component).

**Fig. 8:**
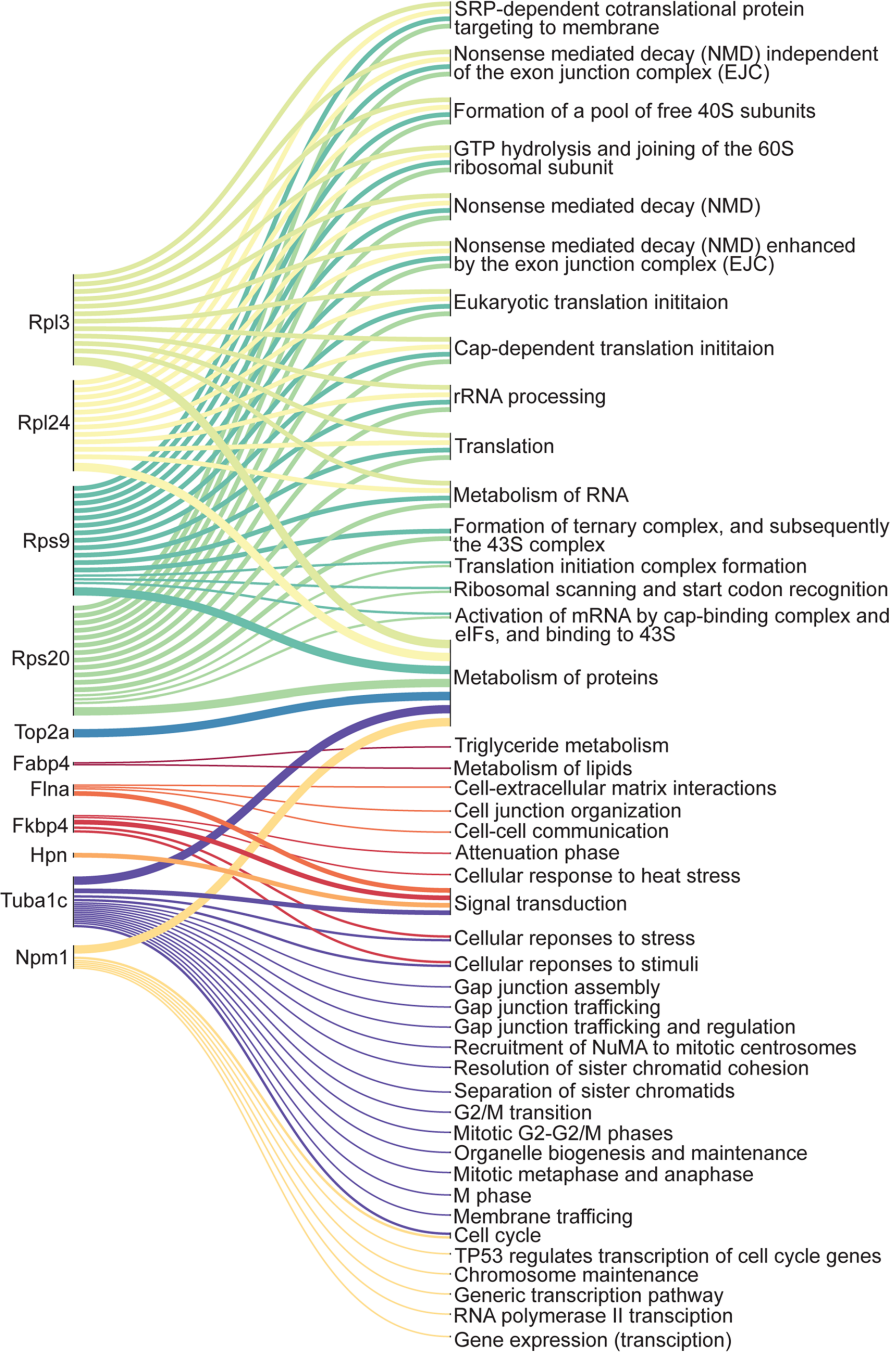
Pathway analysis of the differentially expressed proteins in spermatogonial germ cells isolated from prepubertal mice tissue cryopreserved with CFM and TFM

A list of the pathways analysis using the Reactome database is provided in Supplementary Table 3 and Supplementary Table 4. Pathway analysis of the 29 common proteins indicated SRP-dependent co-translational protein targeting to membrane, nonsense-mediated decay (NMD) independent of the exon junction complex (EJC), nonsense-mediated decay (NMD), nonsense-mediated decay (NMD) enhanced by the exon junction complex (EJC), GTP hydrolysis and joining of the 60S ribosomal subunit, formation of a pool of free 40S subunits, eukaryotic translation initiation, cap-dependent translation initiation due to the significantly higher expression of Rps9, Rpl3, Rpl24 and Rps20 in the TFM and fresh group, compared to CFM (Fig. 8).

## Discussion

In this study, we report the successful formulation of a membrane lipid-based and antioxidant-rich freezing medium optimized for cryopreservation of prepubertal testicular tissue using a slow freezing protocol. The results of the study indicate a significant beneficial effect of this medium over the routinely used DMSO-based freezing medium. The formulation works effectively for adult testicular tissue of mice as well (Supplementary Fig. 4a-c). Further, in this study, we report the freeze–thaw-induced proteomic changes in the prepubertal SGCs and correlate the proteomic profile with the survival of SGCs for the first time.

The freeze–thaw process is known to result in loss of viability in cells of prepubertal testicular tissue (Gouk et al. 2011; Peris-Frau et al. 2023). Efforts to improve the viability by altering the level of FCS (Milazzo et al. 2008; Gouk et al. 2011; Unni et al. 2012; Moraveji et al. 2019), DMSO (Unni et al. 2012; Moraveji et al. 2019) and tissue size (Keros et al. 2007; Wyns et al. 2008; Curaba et al. 2011; Picton et al. 2015), during uncontrolled slow freezing (Baert et al. 2018; Kabiri et al. 2022) have been attempted earlier. Damaged basement membrane, reduction in the number of intact seminiferous tubules, interstitial fibrosis, and decrease in intratubular cell density (Moraveji et al. 2019; Rives-Feraille et al. 2022) are the markers for assessing the consequences of the freeze–thaw process on the testicular architecture. For strategies that depend upon fertility restoration following transplantation techniques, preservation of tissue integrity, intact cell-to-cell communication (Moraveji et al. 2019), and cell viability are crucial (Keros et al. 2007). A decrease in intertubular space, less basal membrane damage, and a twofold higher vimentin expression in the tissues cryopreserved with TFM suggests that the presence of membrane lipids and antioxidants helps in preserving the tissue architecture.

Shedding of phospholipids and cholesterol from the plasma membrane during the freeze–thaw process has been reported earlier (Chakrabarty et al. 2007). The presence of membrane lipid components in the freezing medium is therefore expected to help in preventing the loss of membrane lipids from the cells and/or help the membrane rebuilding process during thawing. The presence of NBD-cholesterol in testicular cells cryopreserved with a freezing medium containing NBD-cholesterol confirmed the cellular uptake of lipids from the freezing medium during the freeze–thaw process (Supplementary Fig. 3). The addition of lecithin (Jeyendran et al. 2008; Reed et al. 2009; Nishijima et al. 2015; Dalmazzo et al. 2018; Zakošek Pipan et al. 2020), cholesterol (Mocé et al. 2010; Behera et al. 2015; Salmon et al. 2016; Lone 2018), phospholipids (Sicchieri et al. 2021) and gangliosides (Gavella et al. 2012) in the freezing medium has shown improvement in the functional competence of frozen-thawed spermatozoa. Further, exogenous supplementation of lipid extract or individual lipid components with or without antioxidants has been observed to be extremely beneficial for larvae cryopreservation (Zhu et al. 2023). The presence of membrane lipids and antioxidants in the medium did not alter the freezing point of the cryopreservation medium (Supplementary Fig. 2) suggesting that the beneficial properties are mainly mediated through preventing the membrane damage and oxidative stress.

Vitamin C is a water-soluble vitamin, which donates electrons and helps in preventing biological macromolecules (lipids, proteins, and DNA) from undergoing oxidation. In the process, once it gets reduced, vitamin C is no longer reactive and fairly stable, making it one of the most preferred antioxidants (Padayatty et al. 2003). Similarly, selenium, a micromineral, exerts its effect by stimulating the expression and activity of antioxidant enzyme glutathione peroxidases in cells to enhance the antioxidant defense system during stressful conditions, facilitating the effective scavenging of lipid hydroperoxides to protect cells from oxidative stress-mediated insult. Presence of potent antioxidants like vitamin C (Thomson et al. 2009; Zini et al. 2009; Branco et al. 2010; Li et al. 2010) and selenium (Rezaeian et al. 2016; Qazi et al. 2019; Nori-Garavand et al. 2020) in the freezing medium are reported to mitigate oxidative stress, thereby improving cryosurvival (Len et al. 2019; Sun et al. 2020). Exogenous supplementation of vitamin C during cryopreservation is reported to reduce oxidative stress thereby preserving DNA integrity and reducing apoptosis (Mangoli et al. 2018; Hungerford et al. 2024). This protection against oxidative stress-induced cellular damage by vitamin C is due to scavenging ROS, vitamin E-dependent neutralization of lipid hydroperoxyl radicals, and protecting proteins from alkylation by lipid peroxidation products (Traber and Stevens 2011). Valadbeygi et al. (2016) observed that supplementation of freezing medium with selenium during cryopreservation improved viability and reduced apoptosis in adipose-derived mesenchymal stem cells. Further, the addition of selenium at low concentrations is reported to significantly reduce the expression of *Bax* and *Caspase-3* during cryopreservation of mice spermatogonial stem cells (Boroujeni et al. 2019) and significantly upregulate *Bcl-2* expression during the vitrification-thawing of mice ovary (Nori-Garavand et al. 2020). The significant decrease in PCO levels observed in the testicular tissues frozen with TFM indicates that the components of the medium help in reducing the oxidative stress generated during cryopreservation. Further, higher activity of selenium-containing GPX isoenzymes that are highly expressed in the testicular tissues (Imai et al. 2009) might have contributed to combating oxidative stress. A significantly higher mRNA expression of *Gpx4* observed in the testicular tissues frozen with TFM confirms this. Earlier studies have shown that cryopreserving cells with selenium significantly reduced oxidative stress in mice spermatogonial stem cells (Boroujeni et al. 2019), adipose-derived mesenchymal stem cells (Valadbeygi et al. 2016), and ovarian tissue of monkeys (Brito et al. 2014). The significantly lower percentage of γ-H_2_AX-positive testicular cells and, lower expression of *Cyt c* and *Caspase-3* mRNA in the tissue frozen with TFM suggest that the incorporation of membrane lipids and antioxidants in the freezing medium prevented freeze–thaw-induced DNA damage and apoptosis.

Cryosusceptibility of testicular cells depends on the species (Peris-Frau et al. 2023), cryopreservation method used (Andrae et al. 2021), proliferation index, free radical scavenging system, and membrane lipid composition (Gironi et al. 2020). In a recent report, Picazo et al. (2022) observed that dog spermatogonial cells, spermatocytes, and elongating spermatids exhibited better survival compared to the elongated spermatids and spermatozoa when cryopreserved by vitrification as well as slow freezing method. Peris-Frau et al. (2023) reported higher viability and DNA integrity of round (spermatogonia, spermatocytes, and early spermatids) cells compared to elongated spermatids and spermatozoa. In our study, we observed that Leydig and germ cells are more vulnerable to freeze–thaw-induced loss of viability compared to Sertoli cells, which agrees with an earlier report by Andrae et al. (2021), who reported a decrease in the number of spermatogonial cells, while no change in the Sertoli cell number per tubule area (µm^2^) after the freeze–thaw process of grey wolf testicular tissue. It has been observed that exosomes, which are lipid-rich (composed of sphingomyelin, phosphatidylserine, cholesterol, and ceramide) (Raposo and Stoorvogel 2013), ameliorate the damaging effects of the freeze–thaw process during canine (Qamar et al. 2019), porcine (Du et al. 2016), and rat (Mokarizadeh et al. 2013) semen cryopreservation (Saadeldin et al. 2020). Similarly, our observation suggests that the presence of membrane lipids and antioxidant molecules in the freezing medium considerably improved the survival of SGCs compared to other cell types. This could be explained by the incorporation of membrane lipids in our freezing medium, which is specific to germ cells (Beckman and Coniglio 1979). However, this needs to be confirmed by further studies.

The freeze–thaw process is reported to cause changes in the cell proteome (Li et al. 2019). However, to the best of our knowledge, the proteomic changes taking place in SGCs following the freeze–thaw process have not been reported so far. In general, freeze–thaw process resulted in alteration in the expression of proteins involved in stress response (Hspa2, Hspa4l, Eef1a1, Hspa2, Fkbp4, Hsp90aa1, Skp1a, Alb, Tuba1c, Eef1a1, Tuba3b, Tubb4b, Tuba3a, Dynll2); DNA damage and apoptosis (Hmgb2, Npm1, Hspa4l, Alb, Tuba1c, Eef1a1, Tuba3b, Tubb4b, Hspa2, Tuba3a, Skp1a, Fkbp4, Dynll2, Hsp90aa1), DNA repair (Rps9, Rpl3, Pabpc1, Rpl24, Rps20, Rpl29, Rpl18, H2afx) and gene regulation (Rps9, Rpl3, Pabpc1, Rpl24, Rps20, Rpl29, Rpl18, Eif3a, Npm1, H2afx, Top2a, Hnrnpu, Tra2b, Srsf1, Hnrnpk, Hnrnpa2b1, Hnrnpl, Skp1a; Supplementary Table 3 and 4).

Among the top 10 upregulated proteins, in the CFM, a 20-fold increase in Cofilin 2 (Cfl2) was observed in SGCs cryopreserved in CFM compared to the fresh samples. Cofilin is a membranous protein, which is known to have a significant role in actin filament dynamics in the cytoplasm (Hurst et al. 2019). In neuronal cells, high expression of cofilin is correlated with p53 translocation to the mitochondria leading to apoptosis (Liu et al. 2017), which supports our finding observed in frozen-thawed SGCs. Further, Nynca et al. (2015) observed a significant increase in Cfl2 expression in the extracellular fluid of cryopreserved rainbow trout semen compared to fresh semen. Further, an increase in the level of other cytoskeleton-associated proteins like Tropomodulin-2 (Tmod2), Tropomyosin-1 (Tpm1), and Krt16 in SGCs of CFM group suggests the freeze–thaw process-induced disruption of the cytoskeletal dynamics. Elevated level of Fatty acid binding protein 4 (FABP4) in SGCs of CFM might be due to the oxidative stress generated during the freeze–thaw process. High FABP4 levels have been documented in conditions of high lipid peroxidation in cells and tissues (Gong et al. 2018; Chen et al. 2023). Similarly, carbonic anhydrase 3 (car3), an early indicator of oxidative damage (Renner et al. 2017), was found to be 11-fold higher in the SGCs of the CFM group indicating freeze–thaw-induced oxidative damage. On the contrary, we observed a high level of SERPINC1 protein in the frozen-thawed SGCs, the knockdown of which has been shown to increase mitochondria-mediated apoptosis (Xu et al. 2019).

The decreased Ybx2, Ldhc, Acyp1, Hspa2, and Nap1l4 levels in the proteome, explain the low survival of SGCs cryopreserved in CFM. Earlier studies have demonstrated that the downregulation of these proteins can increase apoptosis and decrease cell survival (Kleene 2016; Seify et al. 2019; Ma et al. 2021; Sakano et al. 2022). Hist1h1a, a gene that encodes H1.1, which binds to linker DNA between nucleosomes, was observed to be downregulated in CFM. Although the role of histone H1 during the freeze–thaw process is not known in mammalian cells, its role in response to abiotic stress (low temperature, high salinity, drought, and oxidative stress) is studied in plants (Scippa et al. 2000; Rutowicz et al. 2015). Wang et al. (2014) demonstrated that the overexpression of histone H1 in transgenic tobacco plants promoted chromatin condensation and higher tolerance to oxidative and cold-induced stress. Further, Nuclear autoantigenic sperm protein (Nasp), a protein that binds histone H1 was observed to have a role in nucleosome remodeling during DNA repair (Richardson et al. 2006). The low levels of Histone H1 and Nasp observed in SGCs from tissue frozen with CFM may explain the freeze–thaw-induced chromatin alteration and reduced tolerance to cold-induced stress.

The differential proteomic profile of SGCs collected from tissues frozen using TFM supports the higher viability and DNA integrity obtained in TFM compared to those frozen in CFM. Elevated expression of nucleosome proteins (Hist1h1a and Hist1h1c), cytoskeletal proteins (Tpm1, tpm2, Tuba1c, Mylk, and Flna), DNA damage response proteins (Fkbp6, Fkbp4 and Fkbp5), ribosomal proteins (Rsp20, Rpl3, Rps9, and Rpl24), apoptosis proteins (Ybx2 and Top2a), and protein involved with survival (nap1l4) collectively indicate higher chromatin stability, lower cytoskeletal damage, better response to DNA damage and reduction in apoptosis thereby improving the cell survival (pathway analysis; Supplementary Table 3 and 4).

Heat shock proteins are demonstrated to show differential expression during cryopreservation. Qi et al. (2020) demonstrated that the exogenous addition of Hsp90 in the cryopreservation medium prevented oxidative stress-induced damage in rooster spermatozoa. Similarly, in pigs, spermatozoa expressing high levels of Hsp90aa1 were found to be more cryo-resilient (Casas et al. 2010). Earlier studies indicate that Hsp90ab1 functions as a molecular chaperone and exerts a protective effect by binding to proteins, ensuring proper folding and maintaining stability, particularly after exposure to cellular stress (Haase and Fitze 2016). The low levels of Hspa2 in CFM and high Hsp90aa1 in TFM, clearly suggest that the SGCs cryopreserved using TFM are more cryotolerant. The presence of membrane lipids and antioxidants in the freezing medium reduced the apoptosis in SGCs. These results agree with the proteomics data, where a significant increase in levels of Fkbp6, Nap1l4, and Top2a was observed in the TFM. In the literature, knockdown of Fkbp6 is reported to result in increased TUNEL-positive spermatocytes (Crackower et al. 2003), indicating that Fkpb6 levels are essential for germ cell function. Zhu et al. (2022) demonstrated that the knockdown of NAP1L4 in SKOV3 and OVCAR3 cells showed decreased cell proliferation, cell cycle arrest at the G1/S phase, and increased apoptosis. Further, Wu et al. (2023), observed that the miRNA-mediated knockdown of DNA topoisomerase II (TOP2a), increased apoptotic cells, whereas overexpression of TOP2a significantly reduced apoptosis.

Differential expression of ribosomal proteins during the freeze–thaw process (Touil et al. 2023) and their role in adaptation to low temperatures (Jones and Inouye 1996; Wu et al. 2008; Thorne et al. 2020) has been reported earlier. In the current study Rps20, Rpl3, Rps9, and Rpl24 were observed to be overexpressed in TFM and downregulated in CFM. Boeing et al. (2016), have demonstrated that the knockdown of Rsp9 results in elevated γ-H2AX levels in cells exposed to ionizing radiation indicating its protective role in maintaining DNA integrity. These results further suggest that the SGCs in the TFM are more tolerant to cryopreservation-induced changes compared to the CFM.

In conclusion, protecting the cells from freeze–thaw-induced membrane lipid damage and preventing oxidative stress appears to be a meaningful approach in research toward improving the tissue preservation outcome. Considering the mechanism of action of the freezing medium developed, this technology should be extendable to improve the cryopreservation of any other vital organs/ tissues/ cells. We have not tested the beneficial properties of membrane lipids and antioxidant-rich freezing medium on the testicular tissues of prepubertal boys, which is a major limitation of this study. Further, possible differences between mouse and human prepubertal testicular tissue with respect to the outcome of the freeze–thaw process, and the functional ability of the frozen-thawed testicular germ cells in generating the spermatozoa is not tested. However, this study has directed future research in improving the male germ cells, which might eventually lead to the successful generation of functionally competent spermatozoa, suitable for ART (assisted reproductive technology).

## Supplementary Information

Below is the link to the electronic supplementary material.

Supplementary file1 (DOCX 3146 KB)

## Data Availability

MS data are deposited in the PRIDE database under accession code PXD050080. All other data generated or analyzed during this study are included in this published article (and its Supplementary Information files).

## Abbreviations

*CFM*: Control freezing medium
*TFM*: Test freezing medium
*DMSO*: Dimethyl sulfoxide
*SGC*: Spermatogonial germ cells
FBS: Fetal bovine serum
*DMEM/F12*: Dulbecco's Modified Eagle Medium/ Nutrient Mixture F12 Ham
*ROS*: Reactive oxygen species
*PUFAs*: Polyunsaturated fatty acids
*BSA*: Bovine serum albumin
*PFA*: Paraformaldehyde
*TMT*: Tandem mass tag
*FDR*: False discovery rate
*DEPs*: Differentially expressed proteins
*GO*: Gene ontology
*STRING*: Search tool for the retrieval of interacting genes/ proteins
