# Supplementary material for: Enhanced cell survival in prepubertal testicular tissue cryopreserved with membrane lipids and antioxidants rich cryopreservation medium
Source: Cell Tissue Res. 2024 Nov 25;399(1):97–117. doi: 10.1007/s00441-024-03930-6 (PMC11742869; doi:10.1007/s00441-024-03930-6)
Supplement: Supplementary file 1 — Supplementary file1 (DOCX 3146 KB) [file 441_2024_3930_MOESM1_ESM.docx]

**Enhanced Cell Survival in Prepubertal Testicular Tissue Cryopreserved with Membrane Lipids and Antioxidants Rich Cryopreservation Medium**

Reyon Dcunha^1^, Anjana Aravind^2^, Smitha Bhaskar^3^, Sadhana P Mutalik^4^, Srinivas Mutalik^4^, Sneha Guruprasad Kalthur^5^, Anujith Kumar^3^, Padmaraj Hegde^6^, Satish Kumar Adiga^7^, Yulian Zhao^8^, Nagarajan Kannan^9,10,11^, Keshava Prasad Thottethodi Subrahmanya^2^, Guruprasad Kalthur^12#^

^1^Division of Reproductive Genetics, Department of Reproductive Science, Kasturba Medical College, Manipal, Manipal Academy of Higher Education, Manipal- 576104, Karnataka, India

^2^Center for Systems Biology and Molecular Medicine, Yenepoya Research Center, Yenepoya (Deemed to be University), Mangalore- 575018, Karnataka, India

^3^Manipal Institute of Regenerative Medicine, Manipal Academy of Higher Education, Allalasandra, Yelahanka, Bengaluru- 560065, Karnataka, India.

^4^Department of Pharmaceutics, Manipal College of Pharmaceutical Sciences, Manipal Academy of Higher Education, Manipal- 576104, Karnataka, India

^5^Department of Anatomy, Kasturba Medical College, Manipal, Manipal Academy of Higher Education, Manipal- 576104, Karnataka, India

^6^Department of Urology, Kasturba Medical College, Manipal, Manipal Academy of Higher Education, Manipal- 576104, Karnataka, India.

^7^Centre of Excellence in Clinical Embryology, Department of Reproductive Science, Kasturba Medical College, Manipal, Manipal Academy of Higher Education, Manipal- 576104, Karnataka, India

^8^Department of Obstetrics and Gynecology and Department of Laboratory Medicine and Pathology, Mayo Clinic, Rochester- 55905, MN, USA.

^9^Division of Experimental Pathology and Laboratory Medicine, Department of Laboratory

Medicine and Pathology, Mayo Clinic, Rochester- 55905, MN, USA

^10^Center for Regenerative Medicine, Mayo Clinic, Rochester- 55905, MN, USA

^11^Mayo Clinic Cancer Center, Mayo Clinic, Rochester- 55905, MN, USA

^12^Division of Reproductive Biology, Department of Reproductive Science, Kasturba Medical College, Manipal, Manipal Academy of Higher Education, Manipal- 576104, Karnataka, India

**#Corresponding authors:**

Dr Guruprasad Kalthur, MSc, PhD

Division of Reproductive Biology

Department of Reproductive Science

Kasturba Medical College, Manipal,

Manipal Academy of Higher Education, Manipal- 576104, India

Electronic Mail: [guru.kalthur@manipal.edu](mailto:guru.kalthur@manipal.edu)

ORCID ID: 0000-0002-4554-2917

Dr Thottethodi Subrahmanya Keshava Prasad

Center for Systems Biology and Molecular Medicine,

Yenepoya Research Center,

Yenepoya (Deemed to be University),

Mangalore- 575018, India

Electronic Mail: [tskprasad@gmail.com](mailto:tskprasad@gmail.com)

**Effect of individual membrane lipid components on the outcome of pre-pubertal testicular tissue cryopreservation**

The beneficial effect of membrane lipids during prepubertal testicular tissue was explored by supplementing various concentrations of important membrane lipids in the freezing medium.

Prepubertal testicular tissue was frozen using cryopreservation medium (CFM) supplemented with various concentrations (0.5, 1.0, and 2.0 mg/mL) of cholesterol. After storage in LN2 for one week, the tissues were thawed and assessed for cell viability and DNA damage. The freeze-thaw process resulted in a significant decrease in the cell viability of testicular cells (p<0.001). The presence of cholesterol in the freezing medium resulted in marginal improvement in cell viability. When DNA damage was assessed by comet assay, the freeze-thaw process significantly increased the percentage of damaged cells (p<0.001). Although cholesterol reduced the DNA damage, it was statistically not significant.

Similarly, different concentrations of phosphatidylethanolamine, phosphatidylserine, and phosphatidylcholine were supplemented into the freezing medium (CFM) and evaluated for their effect on freeze-thaw outcome. At 1.0 mg/mL concentration of phosphatidylethanolamine marginal improvement in cell viability and reduced DNA damage was observed. Similarly, phosphatidylserine at 0.25 mg/mL concentration marginally improved cell viability and reduced DNA damage. However, phosphatidylcholine did not have any significant beneficial effect on cell viability and DNA damage. Next, different concentrations (0.5, 1.0, 2.5, and 5.0 mg/mL) of soy lecithin were supplemented into the freezing medium. At 2.5 mg/mL concentration, soy lecithin improved the cell viability by 15% and reduced DNA damage in a dose-dependent manner.

The beneficial properties of antioxidant metal ion, sodium selenite on prepubertal testicular tissue cryopreservation were assessed by supplementing different concentrations of sodium selenite (0.001, 0.002, 0.004, and 0.008 mg/mL) to the freezing medium (CFM). The presence of 0.008 mg/mL of sodium selenite in the freezing medium improved cell viability and decreased DNA damage.

The effect of vitamin C, a potent antioxidant was evaluated for the outcome of pre-pubertal testicular tissue cryopreservation by supplementing different concentrations of vitamin C (0.4, 0.6, 0.8 mg/mL) to the freezing medium. At 0.6 mg/mL concentration, vitamin C improved cell viability, reduced DNA damage significantly (p<0.05), and decreased the percentage of apoptotic cells compared to the CFM.

| **Membrane Lipids** | | |
| --- | --- | --- |
| Cholesterol (mg/ mL) | Viability (%) | DNA damage (%) * |
| Fresh tissue | 84.00 ± 6.58 | 5.33 ± 2.08 |
| 0 (CFM) | 41.83 ± 8.97^a^ | 60.00 ± 7.00^a^ |
| 0.5 | 42.50 ± 7.95^a^ | 55.00 ± 3.00^a^ |
| 1.0 | 52.26 ± 2.75^b^ | 52.33 ± 8.14^a^ |
| 2.0 | 47.33 ± 2.56^b^ | 53.33 ± 3.51^a^ |
| Phosphatidylethanolamine (mg/ mL) | Viability (%) | DNA damage (%) * |
| Fresh tissue | 86.60 ± 2.94 | 12.93 ± 3.13 |
| 0 (CFM) | 32.15 ± 1.45^a^ | 45.86 ± 5.39^a^ |
| 0.5 | 30.00 ± 4.27^a^ | 65.73 ± 6.95^a, e^ |
| 1.0 | 41.68 ± 1.93^a^ | 46.66 ± 4.50^a^ |
| 2.5 | 36.72 ± 5.55^a^ | 48.20 ± 3.11^a^ |
| Phosphatidylserine (mg/ mL) | Viability (%) | DNA damage (%) * |
| Fresh tissue | 78.53 ± 5.76 | 5.46 ± 4.52 |
| 0 (CFM) | 30.74 ± 10.02^a^ | 53.30 ± 3.95^a^ |
| 0.1 | 31.78 ± 1.59^a^ | 50.13 ± 5.91^a^ |
| 0.25 | 41.58 ± 6.65^b^ | 44.66 ± 2.85^a^ |
| 0.5 | 29.16 ± 8.97^a^ | 48.30 ± 10.75^a^ |
| 1.0 | 21.35 ± 6.90^a^ | 45.66 ± 3.13^a^ |
| Phosphatidylcholine (mg/ mL) | Viability (%) | DNA damage (%) * |
| Fresh tissue | 86.60 ± 2.94 | 5.8 ± 1.92 |
| 0 (CFM) | 35.27 ± 17.46^c^ | 53.06 ± 2.04^a^ |
| 0.1 | 35.21 ± 8.69^c^ | 49.96 ± 2.45^a^ |
| 0.25 | 32.69 ± 6.15^c^ | 51.56 ± 3.25^a^ |
| 0.5 | 34.93 ± 16.80^c^ | 34.93 ± 16.80^c^ |
| 1.0 | 36.68 ± 23.01^d^ | 36.68 ± 23.01^d^ |
| Soy lecithin (mg/ mL) | Viability (%) | DNA damage (%) * |
| Fresh tissue | 88.83 ± 2.20 | 2.40 ± 1.15 |
| 0 (CFM) | 47.77 ± 6.74^a^ | 37.73 ± 4.59^a^ |
| 0.5 | 50.61 ± 3.41^a^ | 30.16 ± 2.40^a^ |
| 1.0 | 47.70 ± 2.68^a^ | 28.23 ± 2.47^a^ |
| 2.5 | 57.86 ± 2.05^a^ | 25.66 ± 3.30^a, g^ |
| 5.0 | 51.13 ± 4.22^a^ | 25.80 ± 5.65^a, g^ |

**Supplementary Table 1:** Elucidation of optimum concentration of membrane lipid components and antioxidant molecules in control freezing medium for cryopreservation of mouse prepubertal testicular tissue.

| **Antioxidants** | | |
| --- | --- | --- |
| Sodium selenite (mg/ mL) | Viability (%) | DNA damage (%) # |
| Fresh tissue | 82.76 ± 3.01 | 12.76 ± 2.35 |
| 0 (CFM) | 66.98 ± 9.57^d^ | 25.00 ± 2.88^b^ |
| 0.001 | 64.86 ± 2.49^d^ | 21.06 ± 1.89^c^ |
| 0.002 | 72.57 ± 2.98 | 20.86 ± 2.34^d^ |
| 0.004 | 74.42 ± 2.92 | 19.46 ± 2.54^d^ |
| 0.008 | 71.20 ± 0.33 | 23.26 ± 1.70^c^ |
| Vitamin C (mg/ mL) | Viability (%) | DNA damage (%) # |
| Fresh tissue | 83.10 ± 3.87 | 12.93 ± 1.20 |
| 0 (CFM) | 70.80 ± 3.27^c^ | 27.60 ± 1.80^c^ |
| 0.4 | 67.71 ± 2.43^b^ | 21.33 ± 1.41 |
| 0.6 | 75.47 ± 0.63 | 17.13 ± 6.17^f^ |
| 0.8 | 73.59 ± 4.05^d^ | 23.40 ± 2.64^d^ |

The data represents Mean ± SEM. ^a^p< 0.0001, ^b^p< 0.001, ^c^p< 0.01, ^d^p< 0.05 v/s fresh; ^e^p< 0.001, ^f^p<0.01, ^g^p< 0.05 vs 0 (CFM). N=3; results of three independent experiments. *DNA damage in testicular cells was assessed by alkaline comet assay. ^#^DNA damage was assessed by immunostaining testicular cells with anti-γH_2_AX antibody.

**Supplementary Table 2:** List of primers and probes used for qRT-PCR.

| **Gene** | **Primers** | |
| --- | --- | --- |
|  | **Forward primer** | **Reverse primer** |
| *Gapdh* | AGGTCGGTGTGAACGGATTTC | TGTAGACCATGTAGTTGAGGTCA |
| *P53* | GACCGCCGTACAGAAGAAGA | GCGGATCTTGAGGGTGAAATA |
| *Bax* | ATCTGGTTCTGCAAGCGTTTA | CCTGCTCCGAATTTGGTGAAA |
| *Bcl-2* | ATGCCTTTGTGGAACTATATGGC | GGTATGCACCCAGAGTGATGC |
| *Cyt C* | CAGCTTCCATTGCGGACAC | GGCACTCACGGCAGAATGAA |
| *Caspase-3* | ATGGAGAACAACAAAACCTCAGT | TTGCTCCCATGTATGGTCTTTAC |
| **Gene** | **Assay ID of TaqMan® assay probes.** | |
| *Gapdh* | Mm99999915_g1 | |
| *Gpx4* | Mm00515041_m1 | |
| *Catalase* | Mm00437992_m1 | |
| *Sod1* | Mm01344233_g1 | |

**Cell viability assessment in testicular cells from testicular tissue cryopreserved with CFM and TFM**

Immediately after the enzymatic digestion of testicular tissues, the percentage of viable cells was determined by the live and dead cell assay kit (ab115347, Abcam, UK) and trypan blue dye exclusion (Strober 1997). A significant decrease in the percentage of viable cells (p<0.001 and p<0.0001 from live dead assay and trypan blue dye exclusion test respectively) when testicular tissues were cryopreserved using CFM (Supplementary Fig 1a, b). However, cryopreservation using TFM resulted in a non-significantly higher percentage of viable cells compared to CFM.

b

a

**Supplementary Fig. 1:** Viability of testicular cells assessed by using **a** live and dead cell assay kit; and **b** Trypan blue dye exclusion test. The data is represented as Mean ± SEM (N=6).

**Freezing point determination of the CFM and TFM:**

Ice cubes were crushed into small pieces and mixed with sodium chloride (NaCl) to prepare the ice-salt mixture. An alcohol thermometer placed in the ice-salt mixture was used to monitor the temperature below 0°C. To estimate the freezing point of the cryopreservation medium, 2 mL of either CFM or TFM was taken in a test tube and placed in a container with an ice-salt mixture. Precaution was taken to ensure that the medium in the test tubes was below the level of the ice-salt mixture. Milli-Q^®^ (ZR0Q008WW, Merck, USA) water was used as a positive control. The freezing media and MilliQ water froze upon placing them in the ice-salt mixture. As the ice-salt mixture started melting, the temperature of the mixture increased to 0°C. The frozen state of the samples was continuously monitored as the temperature increased using capillary tubes. When the samples turned from solid ice to liquid crystal form, the temperature (freezing point) was recorded. The presence of membrane lipids in the freezing medium resulted in a non-significant increase in the freezing point. The freezing point of the CFM was -8.5 ± 0.51°C, which was higher in TFM (-6.875 ± 0.79°C) (Supplementary Fig. 2).

**Supplementary Fig. 2**: Freezing point of CFM and TFM. The data is represented as Mean ± SEM (N=6).

**Uptake of (NBD)-cholesterol [22-(*N*-(7-Nitrobenz-2-Oxa-1,3-Diazol-4-yl) Amino)-23,24-Bisnor-5-Cholen-3β-Ol] by testicular cells during freeze-thaw process:**

To understand if the testicular tissue uptakes the membrane lipid components during the freeze-thaw process, we added NBD-cholesterol (N1148, Thermo Fisher Scientific, USA) into the freezing medium, and the testicular fragments were subjected to cryopreservation as mentioned earlier. After thawing, the tissues were enzymatically digested using trypsin (1.0 mg/mL) and collagenase (1.0 mg/mL), and the single-cell suspension obtained was passed through a 70 and 40 µm cell strainer. The flow through was centrifuged at 100 x g for 10 min and the obtained cell pellet was resuspended in PBS and observed under a confocal microscope (Zeiss LSM 880, Germany)**.**

The images were captured using Leica TCS SP8 software, Germany**.** The cells that showed fluorescence were counted and expressed as a percentage. All the steps in the procedure were performed in the dark. We observed that 76.88 ± 2.1% of cells were with NBD-cholesterol (Supplementary Fig. 3), which suggests that the cholesterol added into the freezing medium is taken up by the tissue during the freeze-thaw process and incorporated into the membrane.

**
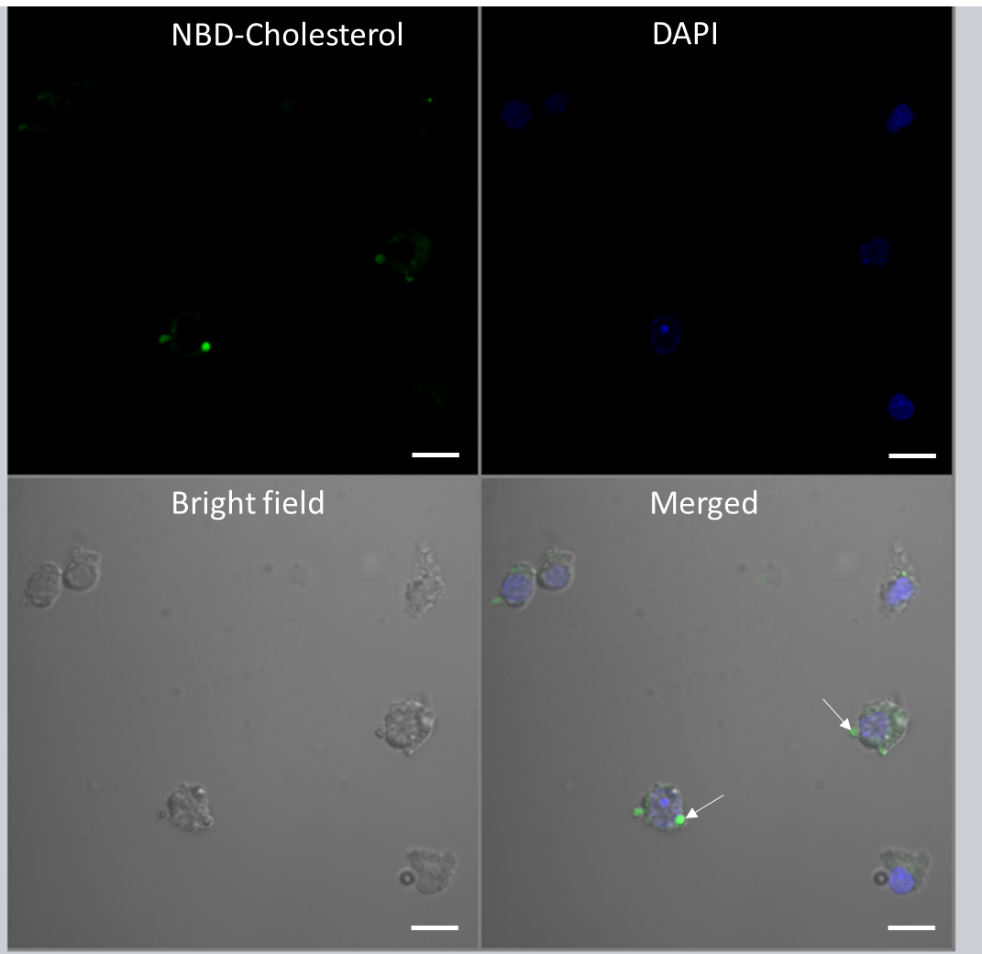
**

**Supplementary Fig. 3:** Uptake of NBD-cholesterol by testicular cells during freeze-thaw process. Arrow indicates NBD-cholesterol. The scale bar represents 10 μm. The data represents three separate trials.

**Effect of freeze-thaw process on adult testicular tissue from Swiss albino mice cryopreserved with CFM or TFM:**

As observed in prepubertal testicular tissue, a significant decrease in viability (P<0.001) was observed in tissue frozen with CFM compared to fresh tissue. The TFM marginally improved the viability (**Supplementary Fig. 4a**) and DNA integrity (**Supplementary Fig. 4b**) compared to tissues frozen in CFM. However, no change was observed in the survival (Supplementary Fig. 4c) of testicular spermatozoa compared to CFM.

c

b

 **Supplementary Fig. 4:** Effect of freeze-thaw process on the outcome in adult testicular tissues of adult Swiss albino mice cryopreserved with CFM or TFM. **a** Viability of testicular cells; **b** DNA damage assessed by γ-H_2_AX expression in testicular cells; and **c** Viability of testicular spermatozoa from adult Swiss albino mice cryopreserved in TFM. The data is represented as Mean ± SEM (N=6).

a

**Validation of MS data**

The proteomic changes in SGCs isolated from fresh, CFM, and TFM groups, were validated by the immunofluorescence technique, as explained earlier. The VASA (DDX4)-positive cells in the fresh and the cryopreserved groups (CFM and TFM) were scored and expressed as a percentage. Compared to the fresh tissue, a significant (p<0.01) reduced number of VASA-positive cells was observed in the cryopreserved group. Although a higher percentage of VASA expression was observed in the TFM compared to control, the increase was non-significant **(Supplementary Fig 5a, b).** This data matches the observation made with the proteomic data.

b

a


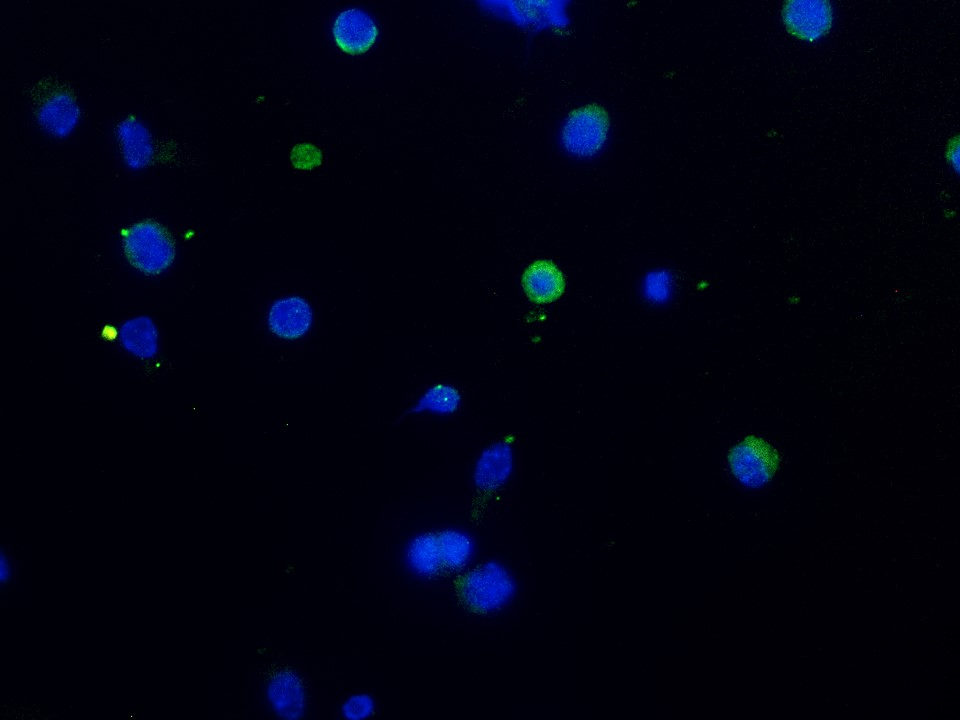

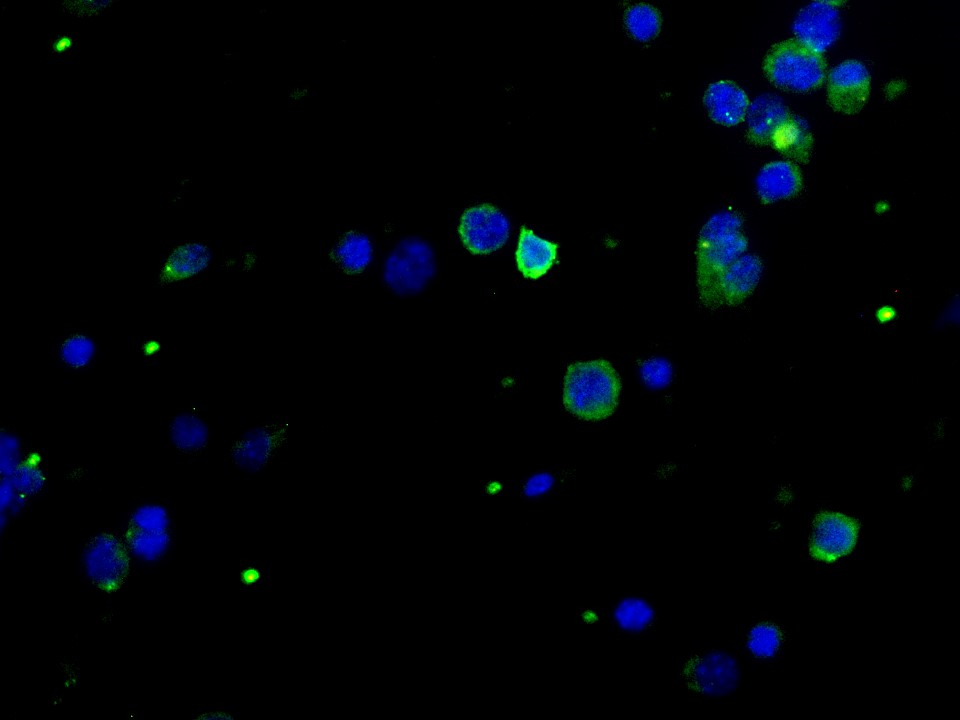

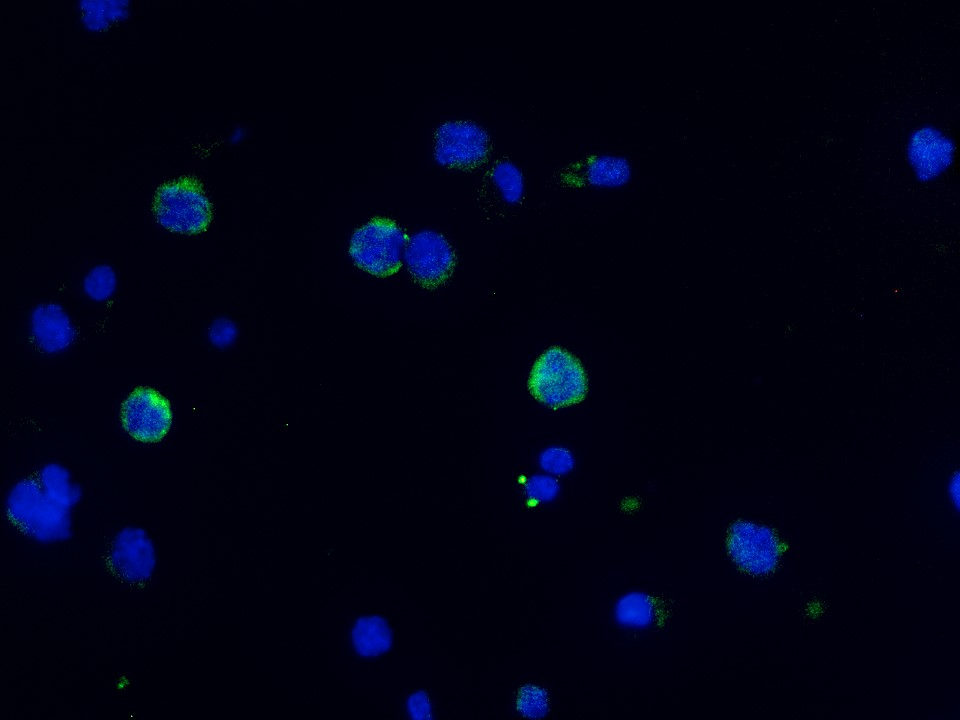


Fresh

CFM

TFM

**Supplementary Fig. 5:** **a** Quantification of VASA (DDX4)-positive SGCs isolated from fresh, CFM, and TFM. **b** Representative merged images for SGCs positive for VASA (DDX4). Nuclear stain DAPI. The data is represented as Mean ± SEM (N=6). The arrow represents VASA (DDX4)-positive cells. The scale bar represents 50 μm.


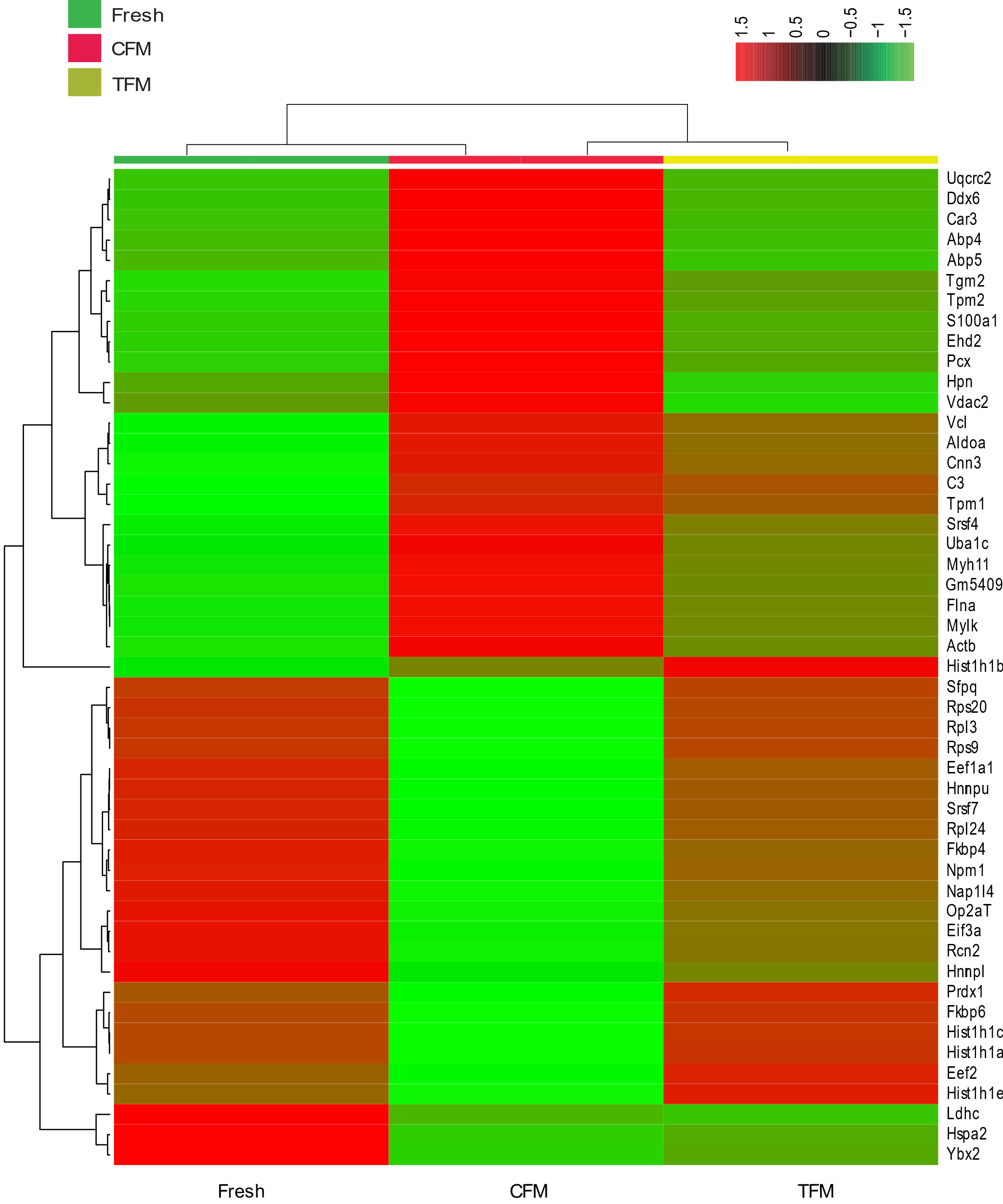


**Supplementary Fig. 6:** Heat map representing the differential expression of proteins from prepubertal mice spermatogonial germ cells (SGCs) isolated from fresh, CFM, and TFM groups. Each row represents a differentially expressed protein and each column represents the mean expression of the protein of three individual trials. The color scale depicts the relative level of protein expression: red, lower than the reference channel; green, higher than the reference.

**Supplementary Table 3:** Pathway analysis of differentially expressed proteins in the CFMs

| **Pathway identifier** | **Pathway name** | **Submitted entities found** |
| --- | --- | --- |
| **DNA repair** | | |
| R-MMU-927802 | Nonsense-Mediated Decay (NMD) | Rps9, Rpl3, Pabpc1, Rpl24, Rps20, Rpl29, Rpl18 |
| R-MMU-5693571 | Nonhomologous End-Joining (NHEJ) | H2afx |
| R-MMU-5693606 | DNA Double Strand Break Response | H2afx |
| R-MMU-69473 | G2/M DNA damage checkpoint | H2afx |
| R-MMU-5693607 | Processing of DNA double-strand break ends | H2afx |
| R-MMU-5693538 | Homology Directed Repair | H2afx |
| R-MMU-73894 | DNA Repair | H2afx |
| R-MMU-5693532 | DNA Double-Strand Break Repair | H2afx |
| **Stress** | | |
| R-MMU-3371511 | HSF1 activation | Eef1a1, Hsp90aa1 |
| R-MMU-3371568 | Attenuation phase | Hspa2, Fkbp4, Hsp90aa1 |
| R-MMU-3371571 | HSF1-dependent transactivation | Hspa2, Fkbp4, Hsp90aa1 |
| R-MMU-3371556 | Cellular response to heat stress | Hspa4l, Eef1a1, Hspa2, Fkbp4, Hsp90aa1 |
| R-MMU-3371453 | Regulation of HSF1-mediated heat shock response | Hspa4l, Hspa2 |
| R-MMU-450294 | MAP kinase activation | Skp1a |
| R-MMU-9755511 | KEAP1-NFE2L2 pathway | Skp1a |
| R-MMU-9711123 | Cellular response to chemical stress | Alb, Skp1a |
| R-MMU-2262752 | Cellular responses to stress | Hspa4l, Alb, Tuba1c, Eef1a1, Tuba3b, Tubb4b, Hspa2,  Tuba3a, Skp1a, Fkbp4, Dynll2, Hsp90aa1 |
| R-MMU-3700989 | Transcriptional Regulation by TP53 | Npm1 |
| **DNA damage and apoptosis** | | |
| R-MMU-140342 | Apoptosis-induced DNA fragmentation | Hmgb2 |
| R-MMU-6791312 | TP53 Regulates Transcription of Cell Cycle Genes | Npm1 |
| R-MMU-109606 | Intrinsic Pathway for Apoptosis | Dynll2 |
| R-MMU-9612973 | Autophagy | Tuba1c, Tuba3b, Tubb4b, Tuba3a, Dynll2 |
| R-MMU-75153 | Apoptotic execution phase | Hmgb2 |
| R-MMU-5357801 | Programmed Cell Death | Hmgb2, Hsp90aa1, Dynll2 |
| R-MMU-174143 | APC/C-mediated degradation of cell cycle proteins | Skp1a |
| R-MMU-3371511 | HSF1 activation | Eef1a1, Hsp90aa1 |
| R-MMU-3371568 | Attenuation phase | Hspa2, Fkbp4, Hsp90aa1 |
| R-MMU-3371571 | HSF1-dependent transactivation | Hspa2, Fkbp4, Hsp90aa1 |
| R-MMU-3371556 | Cellular response to heat stress | Hspa4l, Eef1a1, Hspa2, Fkbp4, Hsp90aa1 |
| R-MMU-3371453 | Regulation of HSF1-mediated heat shock response | Hspa4l, Hspa2 |
| R-MMU-450294 | MAP kinase activation | Skp1a |
| R-MMU-9755511 | KEAP1-NFE2L2 pathway | Skp1a |
| R-MMU-9711123 | Cellular response to chemical stress | Alb, Skp1a |
| R-MMU-2262752 | Cellular responses to stress | Hspa4l, Alb, Tuba1c, Eef1a1, Tuba3b, Tubb4b, Hspa2, Tuba3a, Skp1a, Fkbp4, Dynll2, Hsp90aa1 |
| R-MMU-3700989 | Transcriptional Regulation by TP53 | Npm1 |
| **Gene regulation** | | |
| R-MMU-72613 | Eukaryotic Translation Initiation | Rps9, Rpl3, Pabpc1, Rpl24, Rps20, Rpl29, Rpl18, Eif3a |
| R-MMU-72649 | Translation initiation complex formation | Rps9, Pabpc1, Rps20, Eif3a |
| R-MMU-72662 | Activation of the mRNA upon binding of the cap-binding complex and eIFs, and subsequent binding to 43S | Rps9, Pabpc1, Rps20, Eif3a |
| R-MMU-72662 | Activation of the mRNA upon binding of the cap-binding complex and eIFs, and subsequent binding to 43S | Rps9, Pabpc1, Rps20, Eif3a |
| R-MMU-8864260 | Transcriptional regulation by the AP-2 (TFAP2) family of transcription factors | Npm1 |
| R-MMU-72312 | rRNA processing | Rps9, Rpl3, Ncl, Rpl24, Rps20, Rpl29, Rpl18 |
| R-MMU-774815 | Nucleosome assembly | Npm1, H2afx |
| R-MMU-3899300 | SUMOylation of transcription cofactors | Npm1 |
| R-MMU-72766 | Translation | Rps9, Rpl3, Pabpc1, Eef1a1, Rpl24, Rps20, Rpl29, Rpl18, Eif3a |
| R-MMU-4615885 | SUMOylation of DNA replication proteins | Top2a |
| R-MMU-72172 | mRNA Splicing | Hnrnpu, Tra2b, Srsf1, Hnrnpk, Hnrnpa2b1, Hnrnpl |
| R-MMU-8939902 | Regulation of RUNX2 expression and activity | Skp1a |
| R-MMU-73856 | RNA Polymerase II Transcription Termination | Srsf1 |
| R-MMU-73857 | RNA Polymerase II Transcription | Npm1, H2afx, Srsf1, Skp1a |

**Supplementary Table 4:** Pathway analysis of differentially expressed proteins in the CFMs

| **Pathway identifier** | **Pathway name** | **Submitted entities found** |
| --- | --- | --- |
| **DNA repair** | | |
| R-MMU-927802 | Nonsense-Mediated Decay (NMD) | Rps9, Rpl3, Rpl24, Rps20 |
| R-MMU-5693571 | Nonhomologous End-Joining (NHEJ) | Hist1h4m, Hist4h4, Hist1h4k, Hist2h4, Hist2h2bb, Hist1h4f, Hist1h4c, Hist1h4d, Hist1h4i, Hist1h4j, Hist1h4h, Hist1h4a, Hist1h4b |
| R-MMU-5693565 | Recruitment and ATM-mediated phosphorylation of repair and signaling proteins at DNA double-strand breaks | Hist1h4m, Hist4h4, Hist1h4k, Hist2h4, Hist2h2bb, Hist1h4f, Hist1h4c, Hist1h4d, Hist1h4i, Hist1h4j, Hist1h4h, Hist1h4a, Hist1h4b |
| R-MMU-5693606 | DNA Double Strand Break Response | Hist1h4m, Hist4h4, Hist1h4k, Hist2h4, Hist2h2bb, Hist1h4f, Hist1h4c,  Hist1h4d, Hist1h4i, Hist1h4j, Hist1h4h, Hist1h4a, Hist1h4b |
| R-MMU-73886 | Chromosome Maintenance | Npm1, Hist1h4m, Hist4h4, Hist1h4k, Hist2h4, Hist2h2bb, Hist1h4f, Hist1h4c, Hist1h4d, Hist1h4i, Hist1h4j, Hist1h4h, Hist1h4a, Hist1h4b |
| R-MMU-5693607 | Processing of DNA double-strand break ends | Hist1h4m, Hist4h4, Hist1h4k, Hist2h4, Hist2h2bb, Hist1h4f, Hist1h4c, Hist1h4d, Hist1h4i, Hist1h4j, Hist1h4h, Hist1h4a, Hist1h4b |
| R-MMU-5693567 | HDR through Homologous Recombination (HRR) or Single Strand Annealing (SSA) | Hist1h4m, Hist4h4, Hist1h4k, Hist2h4, Hist2h2bb, Hist1h4f, Hist1h4c, Hist1h4d, Hist1h4i, Hist1h4j, Hist1h4h, Hist1h4a, Hist1h4b |
| R-MMU-5693538 | Homology Directed Repair | Hist1h4m, Hist4h4, Hist1h4k, Hist2h4, Hist2h2bb, Hist1h4f, Hist1h4c, Hist1h4d, Hist1h4i, Hist1h4j, Hist1h4h, Hist1h4a, Hist1h4b |
| R-MMU-5693532 | DNA Double-Strand Break Repair | Hist1h4m, Hist4h4, Hist1h4k, Hist2h4, Hist2h2bb, Hist1h4f, Hist1h4c, Hist1h4d, Hist1h4i, Hist1h4j, Hist1h4h, Hist1h4a, Hist1h4b |
| R-MMU-73894 | DNA Repair | Hist1h4m, Hist4h4, Hist1h4k, Hist2h4, Hist2h2bb, Hist1h4f, Hist1h4c, Hist1h4d, Hist1h4i, Hist1h4j, Hist1h4h, Hist1h4a, Hist1h4b |
| **Stress** | | |
| R-MMU-3371568 | Attenuation phase | Fkbp4, Hsp90ab1 |
| R-MMU-3371571 | HSF1-dependent transactivation | Fkbp4, Hsp90ab1 |
| R-MMU-3371556 | Cellular response to heat stress | Fkbp4, Hsp90ab1 |
| R-MMU-9711123 | Cellular response to chemical stress | Hbb-bs |
| R-MMU-2262752 | Cellular responses to stress | Tuba1c, Hbb-bs, Fkbp4, Hsp90ab1 |
| R-MMU-3700989 | Transcriptional Regulation by TP53 | Npm1 |
| **Gene regulation** | | |
| R-MMU-72613 | Eukaryotic Translation Initiation | Rps9, Rpl3, Rpl24, Rps20 |
| R-MMU-72662 | Activation of the mRNA upon binding of the cap-binding complex and eIFs, and subsequent binding to 43S | Rps9, Rps20 |
| R-MMU-8864260 | Transcriptional regulation by the AP-2 (TFAP2) family of transcription factors | Npm1 |
| R-MMU-72312 | rRNA processing | Rps9, Rpl3, Rpl24, Rps20 |
| R-MMU-212165 | Epigenetic regulation of gene expression | Hist1h4m, Hist4h4, Hist1h4k, Hist2h4, Hist2h2bb, Hist1h4f, Hist1h4c, Hist1h4d, Hist1h4i, Hist1h4j, Hist1h4h, Hist1h4a, Hist1h4b |
| R-MMU-72766 | Translation | Rps9, Rpl3, Rpl24, Rps20 |
| R-MMU-429914 | Deadenylation-dependent mRNA decay | Ddx6 |
| R-MMU-72172 | mRNA Splicing | Crnkl1 |
| R-MMU-212436 | Generic Transcription Pathway | Npm1, Hist1h4m, Hist4h4, Hist1h4k, Hist2h4, Hist2h2bb, Hist1h4f, Hist1h4c, Hist1h4d, Hist1h4i, Hist1h4j, Hist1h4h, Hist1h4a, Hist1h4b |

**Declarations:**

**Ethical Approval:** Testicular tissues were collected from adult Swiss albino male mice (6–8 weeks) maintained at the Central Animal Facility, Manipal Academy of Higher Education, Manipal, India. Prior approval (study number IAEC/KMC/65/2022) from the Institutional Animal Ethical Committee of Kasturba Medical College, Manipal, India was obtained for the current work. The experiments performed in this study were under the guidelines advocated by the institutional and national committee for control and supervision of experiments on animals (CPCSEA), New Delhi, India, and by the ARRIVE guidelines.

**Reference**

Strober W (1997) Trypan Blue Exclusion Test of Cell Viability. Curr Protoc Immunol 21:A.3B.1-A.3B.2. https://doi.org/https://doi.org/10.1002/0471142735.ima03bs21
